# A VioA Variant Activates Antibiotic Streptogramins in the Heterologous Host *Streptomyces* sp. OUC20-O

**DOI:** 10.3390/md23050205

**Published:** 2025-05-11

**Authors:** Jie Shan, Liangguang Yue, Luyao Xu, Runyi Wang, Qingzhou Meng, Jun Feng, Joon-Hee Lee, Ming Lu, Huayue Li

**Affiliations:** 1 Key Laboratory of Marine Drugs, Ministry of Education, School of Medicine and Pharmacy, Ocean University of China, Qingdao 266003, China; 2 State Key Laboratory of Photoelectric Conversion and Utilization of Solar Energy, Qingdao New Energy Shandong Laboratory, Shandong C1 Refinery Engineering Research Center, Qingdao Institute of Bioenergy and Bioprocess Technology, Chinese Academy of Sciences, Qingdao 266101, China; fengjun@qibebt.ac.cn; 3 College of Pharmacy, Pusan National University, Busan 46241, Republic of Korea; joonhee@pusan.ac.kr; 4 Laboratory for Marine Drugs and Bioproducts, Qingdao Marine Science and Technology Center, Qingdao 266237, China

**Keywords:** heterologous expression, marine-derived *Streptomyces*, multidrug-resistant, antibiotic, streptogramin

## Abstract

Heterologous expression of the G231L variant of VioA into 16 strains of marine-derived *Streptomyces*, combined with bioactivity tracking, leads to the activation of seven antibiotic streptogramins (**1**–**7**) in *Streptomyces* sp. OUC20-O. Among these, compound **1**, named linstreptogramin, is a new compound with an unusual linear streptogramin skeleton. The planar structure and stereochemistry of compound **1** were established based on extensive MS and NMR spectroscopic analyses, together with ECD calculations. In the antibacterial activity evaluation, compounds **1**–**4** showed significant growth inhibition against the multidrug-resistant *Enterococcus faecium* CCARM 5203 with MIC values of 0.2–1.6 µg/mL, which are comparable to the positive control vancomycin.

## 1. Introduction

Marine actinomycetes are considered to be one of the richest sources of drug-lead natural products, which is attributed to their unique living environments, such as high pressure, low temperature, and poor nutrient availability [1,2,3]. Strains of the genus *Streptomyces*, widely known as a “drug factory”, have abundant biosynthetic gene clusters (BGCs), which are capable of encoding promising natural products [4,5]. However, a large number of BGCs in *Streptomyces* are orphan, cryptic, or silent under normal culture conditions, which remains as a big barrier for the mining of novel secondary metabolites.

A wide range of genetic-reliant approaches are currently used to activate/regulate silent BGCs in microorganisms [6], with increasing interest in cell–cell communication triggered by signaling molecules [7]. Signaling molecules can be potent elicitors of secondary metabolism in microorganisms. Small molecules produced by a microorganism may act as signaling molecules to regulate gene expression in heterologous hosts at concentrations below a certain threshold, thereby leading to the production of inaccessible secondary metabolites [8,9]. Violapyrones, a class of polyketides encoded by the type III polyketide synthase VioA [10], share a similar chemical skeleton with α-pyrone type photopyrones, which serve as signaling molecules at low nanomolar concentrations [11]. In our previous work, we constructed a G231L variant of VioA (pWLI823), which expresses low levels of violapyrones, and achieved the activation of an unusual 14-membered homodimeric macrodiolide brevidiolide through heterologous expression in the marine-derived *Brevibacterium* sp. 7002-073 strain [12]. It provides potential for the application of the G231L variant of VioA as a tool to activate silent BGCs in heterologous hosts.

**Figure 1:**
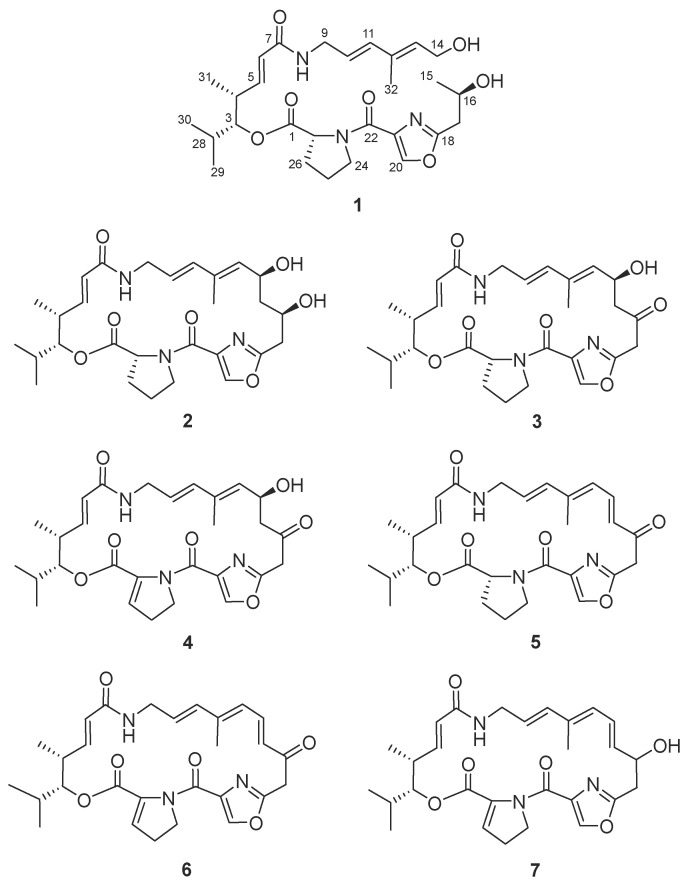
Structures of compounds **1**–**7**.

In this study, the recombinant plasmid pWLI823 was introduced into 16 marine-derived *Streptomyces* strains (Table S6) via conjugation. Combined with antibacterial activity tracking, finding the activation of a series of streptogramin-type compounds (**1**–**7**) (Figure 1) in the recombinant strain of *Streptomyces* sp. OUC20-O/pWLI823. Herein, we describe the heterologous expression-mediated activation, targeted isolation, structure identification, and antibacterial activity of these compounds.

## 2. Results and Discussion

**Figure 2:**
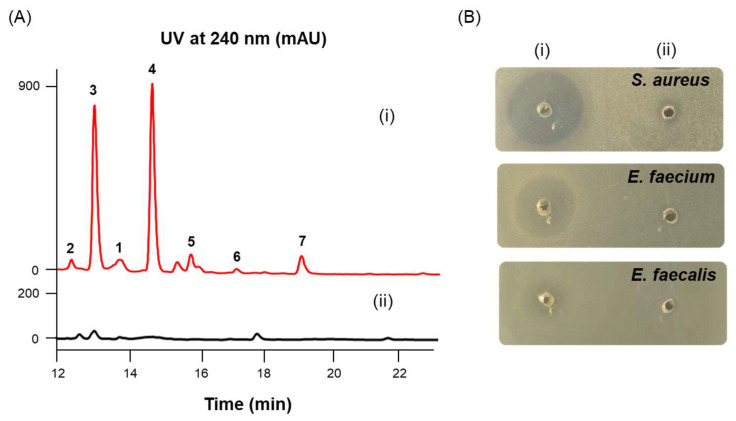
Comparative HPLC (**A**) and antibacterial activity (**B**) analysis of the culture extracts of wild-type and recombinant strains of *Streptomyces* sp. OUC20-O. (**i**) OUC20-O/pWLI823; (**ii**) wild-type OUC20-O. The inhibition zones of the culture extract of the recombinant OUC20-O/pWLI823 strain against the multidrug-resistant (MDR) *Staphylococcus aureus* CCARM 3090, *Enterococcus faecium* CCARM 5203, and *Enterococcus faecalis* CCARM 5172 were 21, 16, and 9 mm, respectively.

We transformed the recombinant plasmid pWLI823, harboring the G231L variant of VioA [12], into 16 marine-derived *Streptomyces* strains, resulting in six recombinant strains being successfully obtained (Figure S1). Subsequently, each recombinant and wild-type strain was fermented and the culture broth was extracted with EtOAc, before being subjected to bioassay-coupled HPLC analysis (Figure S2). In the HPLC profiling of the recombinant strain *Streptomyces* sp. OUC20-O/pWLI823, we observed that a family of secondary metabolites with similar UV-spectra were activated (Figure 2A), which meanwhile showed notably increased inhibitory effects on the multidrug-resistant (MDR) bacterial strains of *Staphylococcus aureus* CCARM 3090, *Enterococcus faecium* CCARM 5203, and *Enterococcus faecalis* CCARM 5172 with inhibition zones of 21, 16 and 9 mm, respectively (Figure 2B). The HPLC profiling of the recombinant strain *Strepomyces* sp. OUCLQ20-1/pWLI823 revealed the activation of a compound, which was identified as rabelomycin based on the UV-HPLC-HRESIMS analysis (Figure S3) [13]; however, it showed no anti-MDR-bacterial activity. The other four recombinant strains (*Strepomyces* sp. OUCYC20-11/pWLI823, *Strepomyces* sp. OUCYC20-13/pWLI823, *Strepomyces* sp. OUCYC20-18/pWLI823, and *Strepomyces* sp. OUCT18-R-3/pWLI823) did not show any metabolic changes compared with their corresponding wild-type strains (Figure S2). Thus, with the purpose of mining new natural antibiotics, the recombinant strain *Streptomyces* sp. OUC20-O/pWLI823 was fermented on a large scale (28 L), followed by the EtOAc extraction, reversed-phase medium-pressure fractionation, and HPLC purification to afford compounds **1**–**7**. The planar and stereochemical structures of these compounds were established based on extensive MS and NMR spectroscopic analyses, Marfey’s reactions, and ECD calculations.

**Figure 3:**
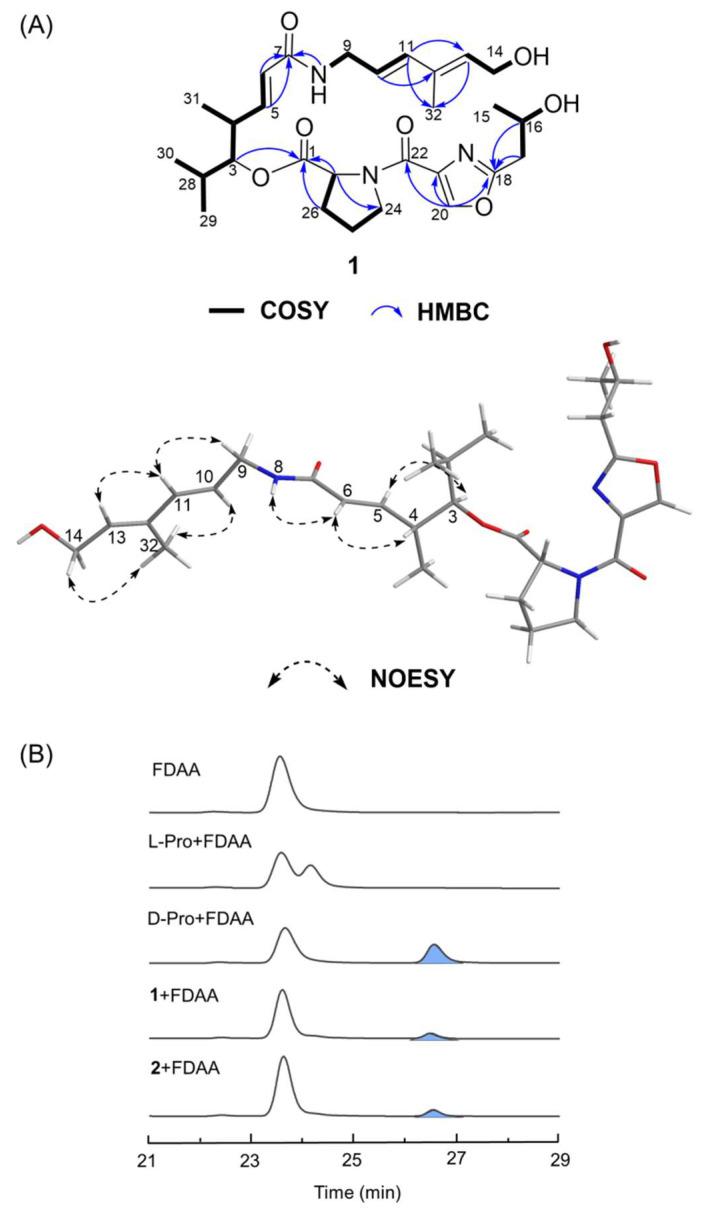
(**A**) COSY, key HMBC, and NOESY correlations of **1**; (**B**) HPLC chromatograms of the FDAA derivatives of standard prolines (Pro) and acid hydrolysates of **1** and **2**, with D-Pro represented by blue peaks.

Compound **1** was obtained as a faint yellow solid with a molecular formula of C_28_H_41_N_3_O_7_ (ten degrees of unsaturation), as deduced by the (+)-HRESIMS ion peak at *m*/*z* 532.3023 [M + H]^+^ (calcd 532.2978). The COSY correlations of H-3/H-4/H-5/H-6/H-28/H-29/H-30/H-31, NH-8/H-9/H-10/H-11, H-13/H-14/14-OH, H-15/H-16/16-OH/H-17, and H-24/H-25/H-26/H-27 revealed five ^1^H spin systems (Figure 3A). The presence of proline (Pro) was determined based on the HMBC correlations from H-27 (δ_H_ 4.49) to C-1 (δ_C_ 172.3) and C-24 (δ_C_ 47.2), and from H-26 (δ_H_ 1.81) to C-1. Based on the HMBC correlations from H-3 to C-1, we determined the connection between the Pro residue and the alkyl fragment. The presence of the oxazole ring was determined by the HMBC correlations from the olefinic proton H-20 (δ_H_ 8.54) to the quaternary carbons C-18 (δ_C_ 162.4) and C-21 (δ_C_ 143.8). The HMBC correlations from H-20 to C-22 (δ_C_ 162.6) and from H-17 (δ_H_ 2.71) to C-18 revealed the connection of the oxazole ring to C-17 (δ_C_ 37.4) and C-22, respectively. Then, combining the HMBC correlations from H-5 (δ_H_ 6.53), H-6 (δ_H_ 5.95), and NH-8 (δ_H_ 8.12) to the amide carbon C-7 (δ_C_ 164.8), we confirmed the connection from C-6 (δ_C_ 124.8) to C-9 (δ_C_ 135.5). The HMBC correlations from H-11 (δ_H_ 6.15), H-13 (δH 5.53) to C-32 (δ_C_ 12.3), and H-10 (δ_H_ 5.57) to C-12 (δ_C_ 133.0) suggested the substitution of a methyl group at C-13. The NMR assignment of **1** demonstrated that its structure is similar to 16-hydroxy-virginiamycin M2 (**2**) (Figure 1), a type-A streptogramin, except for the hydroxylated methine CH-14 (δ_H_ 4.90, δ_C_ 65.4) and the methylene CH_2_-15 (δ_H_ 3.05, 2.88, δ_C_ 48.6) in **2** being converted to a terminal hydroxy methylene (δ_H_ 4.07, δ_C_ 57.5) and a methyl group (δ_H_ 1.12, δ_C_ 23.2) in **1**, respectively. This provided evidence that the C-C bond between C-14 and C-15 is broken in **1**, resulting in a new linear streptogramin skeleton. Notably, the ring-opening site of **1** occurred at a C-C bond rather than the ester linkage. Since such C-C bond cleavage is unprecedented during the type-A streptogramin bioassembly process, we postulate that this bond scission likely occurred post-cyclization, potentially mediated by an unidentified enzyme. The ^1^H and ^13^C chemical shifts of **1** are listed in Table 1.

**Figure 4:**
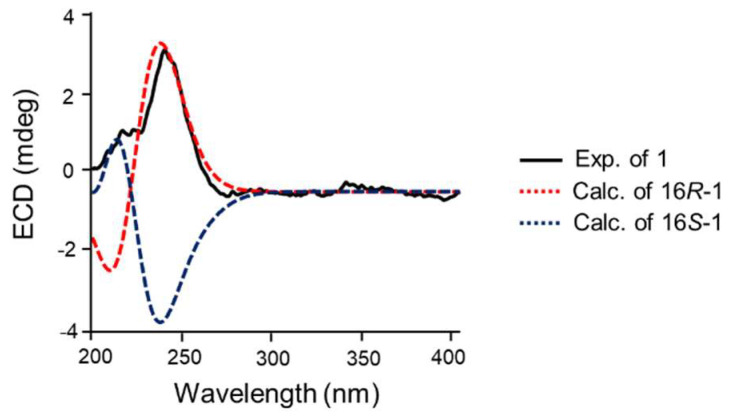
Experimental and calculated ECD spectra of **1**.

The geometrical configurations of the double bonds C-5/C-6, C-10/C-11, and C-12/C-13 of **1** were identified to be *E* by the NOESY correlations of H-6/H-4, H-5/H-3, H-9/H-11, H-10/H-32, H-11/H-13, and H-14/H-32 (Figure 3A). The Pro residue was determined to be the D-form according to Marfey’s analysis (Figure 3B). As **1** bears a linear streptogramin skeleton, the absolute configurations of C-3 and C-4 are considered to be *R*, depending on the biocatalytic preference of the ketoreductase (KR) domain during the biosynthesis of streptogramin-type compounds [14,15]. To establish the absolute configuration of C-16 of **1**, DP4 NMR calculations were conducted using the GIAO method at the mPW1PW91/6-311G(d) level of theory, in which the 16*R*-stereoisomer showed 100% probability (Table 2 and Table S2). Moreover, the ECD curve of 16*R*-**1** calculated at the CAM-B3LYP/6-31G(d) theory level also agreed well with the experimental ECD spectrum of **1** (Figure 4). Thus, the absolute configurations of **1** were established as 3*R*, 4*R*, 16*R*, and 27*R*.

Compounds **2**–**7** were pale yellow amorphous solids. The HRESIMS analysis showed that the molecular ion peaks of **2**–**7** were located at *m*/*z* 530.2874 [M + H]^+^, 526.2565 [M – H]^−^, 526.2543 [M + H]^+^, 510.2599 [M + H]^+^, 508.2444 [M + H]^+^, and 510.2598 [M + H]^+^, respectively. Compound **2**, which was first discovered from a natural source, was determined to be 16-hydroxy-virginiamycin M2 (**1**) by NMR data assignments (Table S1). The Pro residue in **2** was identified to be D-Pro by Marfey’s analysis (Figure 3B). Consistent with **1**, the result of the DP4 calculation of **2** supported the 16*R*-configuration (Table 2 and Table S3). Compounds **3**–**7** were identified as virginiamycin M2 [16,17], virginiamycin M1 [16,17], 14,15-anhydro-virginiamycin M2 [18,19], L-156,587 [20,21], and L-156,586 [20], respectively, by comparing their ^1^H NMR and MS data with those previously reported.

In our investigation of the antibacterial activity of **1**–**7** against Gram-positive MDR strains, compounds **1** and **2** exhibited notable activity against *E. faecium*, with an MIC value of 1.6 μg/mL, which was comparable to the positive control vancomycin (MIC 1.6 μg/mL). Compounds **3** and **4** showed much stronger growth inhibition against *E. faecium* (MICs 0.19 μg/mL), and **4** also showed comparable inhibitory effects on *S. aureus* (MIC 3.1 μg/mL) with that of vancomycin (MIC 3.1 μg/mL). All the compounds were inactive against *E. faecalis* up to 25 μg/mL (Table 3). These results suggest that the hydroxy group at C-14, together with the carbonyl group at C-16, is essential for the antibacterial activity of type-A streptogramins, which is consistent with the previously reported literature [22]. In the cytotoxicity evaluation, the unusual linear-type streptogramin, linsreptogramin (**1**), exhibited very low toxicity to human normal liver cells (L-02) and kidney cells (293T) at 20 μg/mL (Table S4), suggesting its potential utility as an antibiotic drug scaffold.

## 3. Materials and Methods

### 3.1. General Experimental Procedures

Optical rotations were measured on a JASCO P-1030 digital polarimeter (JASCO Corporation, Tokyo, Japan). The ECD spectra in MeOH were obtained using a JASCO J-715 spectropolarimeter. The IR spectra were determined on a Nicolet NEXUE470 FT-IR. 1D and 2D NMR spectra were provided by a Bruker Avance III 600 spectrometer and chemical shifts were reported with reference to the residual solvent peaks (δ_H_ 3.31 and δ_C_ 49.0 for CD_3_OD, δ_H_ 7.26 and δ_C_ 77.1 for CDCl_3_, δ_H_ 3.50 and δ_C_ 39.5 for DMSO-*d*_6_). High-performance liquid chromatography (HPLC) was performed on an Agilent 1360 Infinity equipped with a diode array detector. The HPLC-HRESIMS data were obtained on an Agilent 1260 HPLC system coupled with a Q-TOF Ultima Global GAA076 MS spectrometer. The optical density (OD) measurements were recorded on a Biotech Epoch3 microplate reader.

### 3.2. Transformation Procedures

The spores of each strain were washed twice and resuspended in 1 mL of TSB-Y broth (103 g sucrose, 0.5 g yeast extract, 30 g tryptic soya broth, 1 g tryptone, 1 L H_2_O) and incubated at 50 °C for 10 min. *E. coli* was grown in LB (50 mL) to an OD_600_ of 0.4–0.6 at 37 °C, washed (2 × 40 mL) and resuspended in LB (500 μL). *E. coli* (10 μL) was added to the spores (500 μL), mixed thoroughly by inversion. The mixed liquid was spread on MS agar plates supplemented with 20 mM MgCl_2_. Plates were incubated for 2–3 days at 30 °C. An aliquot of a solution containing nalidixic acid (25 μL) and the appropriate antibiotic (15 μL) for plasmid selection were added to the surface of a petri dish and spread using a glass rod. The plates were then incubated for 5–6 days at 30 °C. The success of the transformation was verified by PCR. The primers required for PCR validation are shown in Table S5.

### 3.3. 16S rRNA Gene Sequencing and Phylogenetic Analysis

All the strains were grown on an MS agar medium (3% soybean meal, 2% mannitol, and 2% agar) at 30 °C for 4–7 days. The mycelia were collected and mixed with a small amount of quartz sand and 0.5 mL of DNA extraction buffer. After incubation at 65 °C for 10 min, 200 μL of 7.5 mol/L ammonium acetate was added and thoroughly mixed, followed by incubation on ice for 8 min. The sample was then centrifuged at 13,000 rpm for 15 min. The supernatant was collected and mixed with ice-cold isopropanol, followed by incubation at −20 °C for 10 min. After centrifugation, the pellet was washed with 70% ethanol, and the DNA was finally dissolved in double-distilled water. The 16S rRNA gene was amplified using universal primers 27F and 1492R (Table S5). The obtained sequence was compared with the 16S rRNA gene sequences of properly classified species in the EzBioCloud database (http://www.ezbiocloud.net/, accessed on 8 May 2025). A phylogenetic tree (Figure S4) was constructed by multiple alignments of the sequence data using the MEGA-X software package.

### 3.4. Fermentation, Extraction, and Isolation

The recombinant strain *Streptomyces* sp. OUC20-O/pWLI823 was first inoculated into 250 mL conical flasks containing 50 mL of M8 broth (2% soluble starch, 1% glucose, 0.2% meat extract, 0.2% yeast extract, 0.3% CaCO_3_ and 0.4% hydrolyzed casein, pH 7.0) and deposited on a rotary oscillator for 7 days at 180 rpm, 30 °C. The combined culture broth (28 L) was extracted with a double volume of EtOAc at room temperature and concentrated by vacuum evaporator. The EtOAc extracts were concentrated (6.4 g) and then partitioned between 90% MeOH and n-hexane to remove non-polar components. The MeOH layer was eluted by MPLC with a gradient of 10–100% MeOH to give 24 fractions (Fr.1–Fr.24). The fractions were further separated by semi-preparative HPLC on a YMC-Pack ODS-A column (250 × 10 mm, 5 μm; flow rate: 1.5 mL/min). Compounds **2** (2.4 mg) and **3** (4.1 mg) were isolated from Fr.13 by isocratic elution with 32% ACN. Compounds **1** (1.0 mg), **4** (3.4 mg), and **5** (5.5 mg) were obtained from Fr.17 using isocratic elution with 35% ACN. Compounds **6** (2.1 mg) and **7** (1.9 mg) were obtained from Fr.19 with 45% ACN.

### 3.5. Marfey’s Analysis

A portion of compound (100 μg) was hydrolyzed with 6 N HCl for 24 h at 110 °C. The hydrolysate was dissolved in 100 μL of H_2_O, and 200 μL of Marfey’s reagent (Nα-(2,4-dinitro-5-fluorophenyl)-L-alaninamide, L-FDAA; 1 mg/mL in acetone) and 50 μL of 1 M NaHCO_3_ were added. Then, the reaction mixture was incubated at 50 °C for 60 min. After cooling to room temperature, 25 μL of 2 N HCl was added to quench the reaction. L- and D-standard amino acids were prepared in the same way as the compounds. Marfey’s reaction products with hydrolysates and standard amino acids were analyzed by HPLC using a ZORBAX SB-C18 (4.6 × 150 mm, 5 μm) column with gradient elution of solvent A (H_2_O + 0.1% TFA) and solvent B (90% ACN + 0.1% TFA) (flow rate: 1 mL/min; wavelength: 340 nm).

### 3.6. Computational Methods

Each stereoisomer was subjected to geometry optimization at the B97D/TZVP level using the Gaussian 09 program. The TD calculations were performed on the optimized conformers using the long-range-corrected hybrid CAM-B3LYP. The number of excited states per molecule was 50. Solvent effects were considered by using the polarizable continuum model (PCM) for methanol. The ECD spectra were generated by the program GaussView 5.0. The NMR chemical shift calculations were managed by the PyDP4 Python 2.7 script within DP4-AI that is available from http://www-jmg.ch.cam.ac.uk/tools/nmr/ (accessed on 8 May 2025) and GitHub https://github.com/KristapsE/DP4-AI/ (accessed on 8 May 2025). The raw ^1^H and ^13^C NMR spectra of compounds **1** and **2**, and a set of 16*S*- and 16*R*-stereoisomers for each compound were prepared as input files. Each conformer was conducted to molecular mechanics calculations in the gas phase utilizing the MMFF force field, which was set to find all low-energy conformers within 10 kcal/mol at least 5 times. The NMR calculations were carried out with the GIAO method at the mPW1PW91/6-311G(d) level of theory.

### 3.7. Radial Diffusion Assay

The multidrug-resistant (MDR) bacterial strains of *S. aureus* CCARM 3090, *E. faecium* CCARM 5303, and *E. faecalis* CCARM 5173 were purchased from the Culture Collection of Antimicrobial Resistant Microbes (Seoul Women’s University of Korea). The strains were grown in LB (10 g peptone, 5 g yeast extract, and 5 g NaCl, 1 L H_2_O) or BHI (Brain Heart Infusion, 38.5 g, 1 L H_2_O) broth at 37 °C overnight and diluted to 5 × 10^5^ CFU/mL. Then, 1 mL of the diluted bacterial culture broth was added to 50 mL of LB/BHI agar medium at 40–50 °C. Once the bacteria were adequately dispersed, the gel was poured into a plate (90 × 90 mm). After solidification, wells were made using a 2 mm punch. Each sample (10 mg/mL, 10 μL) was added to the well, and the plates were incubated at 37 °C for 18 h. Vancomycin (3 mg/mL, 10 μL) was used as a positive control. MeOH was used as a negative control. The diameters of the inhibition zones surrounding the wells were measured in millimeters.

### 3.8. Minimum Inhibitory Concentration (MIC) Assay

Minimum inhibitory concentrations of the compounds against MDR bacterial strains of *S. aureus* CCARM 3090, *E. faecium* CCARM 5303, and *E. faecalis* CCARM 5173 were determined using the broth microdilution method. The strains were grown in LB or BHI broth at 37 °C overnight, and the inoculum was standardized to 5 × 10^5^ CFU/mL. The two-fold serial dilution of the compounds were obtained with 25–0.1 μg/mL in MeOH. After that, we added 20 μL of sample solutions of different concentrations to a 180 μL bacterial suspension in the 96-well plate. MeOH was used as a negative control, and vancomycin was used as a positive control. LB and BHI broth were used as blanks. Then, the plates were incubated at 37 °C for 18 h, and the MIC values were recorded as the lowest concentration of the compounds, in which no visible microbial growths were observed. Each experiment was performed in triplicate. The growth of the MDR strains was measured on a microplate reader at a wavelength of 600 nm.

### 3.9. Cytotoxicity Assay

The cells L-02 (human hepatocytes) and 293T (human embryonic kidney cells) were cultured in 10% fetal bovine serum medium and prepared as single-cell suspensions. In a 96-well plate, 90 μL of cell culture medium (5 × 10^4^/mL for adherent cells and 9 × 10^4^/mL for suspension cells) and 10 μL of sample solution were added, before being incubated at 5% CO_2_ and 37 °C for 24 h. One concentration was set for each sample and three parallels for each concentration. The 96-well plates were cultured at 5% CO_2_ and 37 °C for 48 h. Then, the old medium and drug solution of the adherent cells were aspirated and 100 μL of CCK-8 solution diluted 10-fold with alkaline medium was added, and 10 μL of CCK-8 stock solution was added directly to the suspended cells. Culture was conducted at 37 °C with 5% CO_2_ for 1–4 h (dark operation, real-time observation). The absorbance was measured at 450 nm with an enzyme-labeling instrument and the original data and results were recorded. The toxicity was expressed by cell inhibition, with the following calculation formula: Cell inhibition (%) = (OD_Control_ − OD_Drug_)/(OD_Control_ − OD_Blank_) × 100%. The experimental results were expressed in ±SD.

## 4. Conclusions

In summary, through the bioassay-coupled heterologous expression of the recombinant plasmid pWLI823, into 16 marine-derived *Streptomyces* strains, we activated seven streptogramin-type antibiotics (**1**–**7**) in the recombinant strain *Streptomyces* sp. OUC20-O/pWLI823. Among these, compound **1** features an unusual linear streptogramin skeleton, thus being named as linstreptogramin. Compounds **1**–**7** exhibited significant antibacterial activities against the MDR strain of *E. faecium* (MICs 0.2–1.6 μg/mL), which were comparable (even stronger) to the positive control vancomycin. This study provides a new opportunity for the activation of cryptic compounds from marine *Streptomyces*, and may also be used as an effective tool for the mining of antibiotic lead compounds in microorganisms.

## Figures and Tables

**Table 1:** ^1^H (600 MHz) and ^13^C (150 MHz) NMR data of **1** in DMSO-*d*_6_.

1		
No.	δ_C_, Type	δ_H_ (*J* in Hz)
1	171.8, C	-
3	80.0, CH	4.67, dd (12.7, 6.3)
4	37.2, CH	2.61, m
5	143.9. CH	6.55, td (15.4, 7.5)
6	124.5, CH	5.95, dd (15.5, 7.6)
7	164.8, C	-
8-NH	-	8.10, t
9	40.3, CH_2_	3.80, m
10	125.0, CH	5.57, dt (15.8, 6.5)
11	135.2, CH	6.15, d (15.7)
12	133.0, C	-
13	132.7, CH	5.53, t (6.4)
14	57.5, CH_2_	4.07, m
15	23.2, CH_3_	1.12, d (6.3)
16	64.7, CH	4.04, m
17	37.4, CH_2_	2.71, m
		2.82, d (6.4)
18	162.4, C	-
20	143.9, CH	8.53, m
21	136.2, C	-
22	162.6, C	-
24	48.2, CH_2_	3.91, m
25	24.7, CH_2_	1.92, m
26	28.5, CH_2_	1.81, m
		2.21, m
27	59.4, CH	4.49, dd (8.5, 4.8)
28	29.7, CH	1.82, m
29	19.2, CH_3_	0.87, m
30	17.0, CH_3_	0.85, m
31	13.9, CH_3_	0.94, m
32	12.3, CH_3_	1.67, s
14-OH	-	4.62, t (5.5)
16-OH	-	4.86, dd (12.5, 4.9)

**Table 2:** DP4 NMR Calculations and Probabilities.

Stereoisomer	Low-Energy Conformations ^[a]^	DP4 Probabilities ^[b]^ (%)
16*R*-**1**	61	100
16*S*-**1**	88	0
16*R*-**2**	26	100
16*S*-**2**	4	0

^[a]^ Number of low-energy (<10 kJ/mol) conformations from molecular mechanics calculations. ^[b]^ Probabilities of the calculated NMR data of the stereoisomers using DP4 from comparison of the experimental NMR data.

**Table 3:** Antibacterial activity of compounds **1**–**7**.

Bacterial Strain	Minimum Inhibitory Concentration (MIC) (μg/mL)							
	1	2	3	4	5	6	7	Vancomycin
*S. aureus* CCARM 3090	>25	>25	>25	3.1	>25	>25	>25	3.1
*E. faecium* CCARM 5203	1.6	1.6	0.2	0.2	>25	>25	>25	1.6
*E. faecalis* CCARM 5172	>25	>25	>25	>25	>25	>25	>25	6.2

## Data Availability

The data presented in this study are available upon request from the corresponding author.

## Supplementary Materials

The following supporting information can be downloaded at: https://www.mdpi.com/article/10.3390/md23050205/s1, UV, ECD, IR, HRESIMS and NMR spectra of compounds **1**–**7**; DP4 calculated NMR data of compounds **1** and **2**; cytotoxicity of compound **1**; primer pairs and strains used in the study.
