# Supplementary material for: A VioA Variant Activates Antibiotic Streptogramins in the Heterologous Host Streptomyces sp. OUC20-O
Source: Mar Drugs. 2025 May 11;23(5):205. doi: 10.3390/md23050205 (PMC12113368; doi:10.3390/md23050205)
Supplement: Supplementary file 1 [file marinedrugs-23-00205-s001.zip › marinedrugs-3593469-supplementary.pdf]

Supporting Information

A VioA Variant Activates Antibiotic Streptogramins in the Heterologous Host *Streptomyces* sp. OUC20-O

Jie Shan [1], Liangguang Yue [1], Luyao Xu [1], Runyi Wang [1], Qingzhou Meng [1], Jun Feng [2], Joon-Hee Lee [3], Ming Lu [2,*] and Huayue Li [1,4,*]

[1] Key Laboratory of Marine Drugs, Ministry of Education, School of Medicine and Pharmacy, Ocean University of China, Qingdao 266003, China

[2] State Key Laboratory of Photoelectric Conversion and Utilization of Solar Energy, Qingdao New Energy Shandong Laboratory, Shandong C1 Refinery Engineering Research Center, Qingdao Institute of Bioenergy and Bioprocess Technology, Chinese Academy of Sciences, Qingdao 266101, China

[3] College of Pharmacy, Pusan National University, Busan 46241, Republic of Korea

[4] Laboratory for Marine Drugs and Bioproducts, Qingdao Marine Science and Technology Center, Qingdao 266237, China

Table of contents

Figure S1. PCR confirmation of six recombinant strains of *Streptomyces* spp.

Figure S2. HPLC analysis of the culture extracts of six wild-type and recombinant strains of *Streptomyces* spp.

Figure S3. UV-HPLC-HRESIMS analysis of the activated rabelomycin in the recombinant strain of *Streptomyces* sp. OUCLQ20-1/pWLI823.

Figure S4. Neighbor joining phylogenetic tree based on 16S rRNA sequence of the OUC20-O strain.

Figure S5. UV spectra of **1**−**7** in MeOH.

Figure S6. IR (KBr) spectrum of **1**.

Figure S7. HRESIMS spectrum of **1**.

Figure S8. ^{1}H NMR spectrum of **1** in DMSO-d_6 (600 MHz).

Figure S9. HSQC spectrum of **1** in DMSO-d_6 (600 MHz).

Figure S10. COSY spectrum of **1** in DMSO-d_6 (600 MHz).

Figure S11. HMBC spectrum of **1** in DMSO-d_6 (600 MHz).

Figure S12. NOESY spectrum of **1** in DMSO-d_6 (600 MHz).

Figure S13. HRESIMS spectrum of **2**.

Figure S14. ^{1}H NMR spectrum of **2** in DMSO-d_6 (600 MHz).

Figure S15. HSQC spectrum of **2** in DMSO-d_6 (600 MHz).

Figure S16. COSY spectrum of **2** in DMSO-d_6 (600 MHz).

Figure S17. HMBC spectrum of **2** in DMSO-d_6 (600 MHz).

Figure S18. NOESY spectrum of **2** in DMSO-d_6 (600 MHz).

Figure S19. ^{1}H NMR spectrum of **2** in $CDCl_3$ (500 MHz).

Figure S20. HRESIMS spectrum of **3**.

Figure S21. ^{1}H NMR spectrum of **3** in $CDCl_3$ (500 MHz).

Figure S22. HRESIMS spectrum of **4**.

Figure S23. ^{1}H NMR spectrum of **4** in $CDCl_3$ (500 MHz).

Figure S24. HRESIMS spectrum of **5**.

Figure S25. ^{1}H NMR spectrum of **5** in CD_3OD (500 MHz).

Figure S26. HRESIMS spectrum of **6**.

Figure S27. ^{1}H NMR spectrum of **6** in $CDCl_3$ (500 MHz).

Figure S28. HRESIMS spectrum of **7**.

Figure S29. ^{1}H NMR spectrum of **7** in $CDCl_3$ (500 MHz).

Table S1. ^{1}H (600 MHz) and ^{13}C (150 MHz) NMR data of **2** DMSO-d_6.

Table S2. DP4 calculated ^{1}H NMR data of **1**.

Table S3. DP4 calculated ^{1}H NMR data of **2**.

Table S4. Cytotoxicity of compound **1** toward human normal cells.

Table S5. Primer pairs used in this study.

Table S6. Strains used in this study.

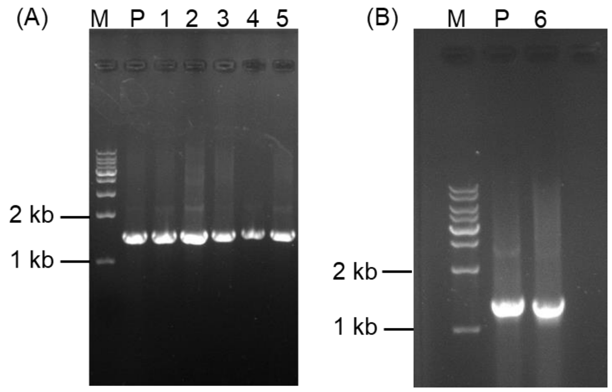

Figure S1. PCR confirmation of six recombinant strains of *Streptomyces* spp. (A) Lane M: 1 kb DNA marker; Lane P: pWLI823 (positive control); Lane 1: OUCLQ20-1/pWLI823; Lane 2: OUCY20-11/pWLI823; Lane 3: OUCY20-13/pWLI823; Lane 4: OUCYC20-18/pWLI823; Lane 5: OUCCT18-R-3/pWLI823. (B) Lane M: 1 kb DNA marker; Lane P: pWLI823 (positive control); Lane 6: OUC20-O/pWLI823.

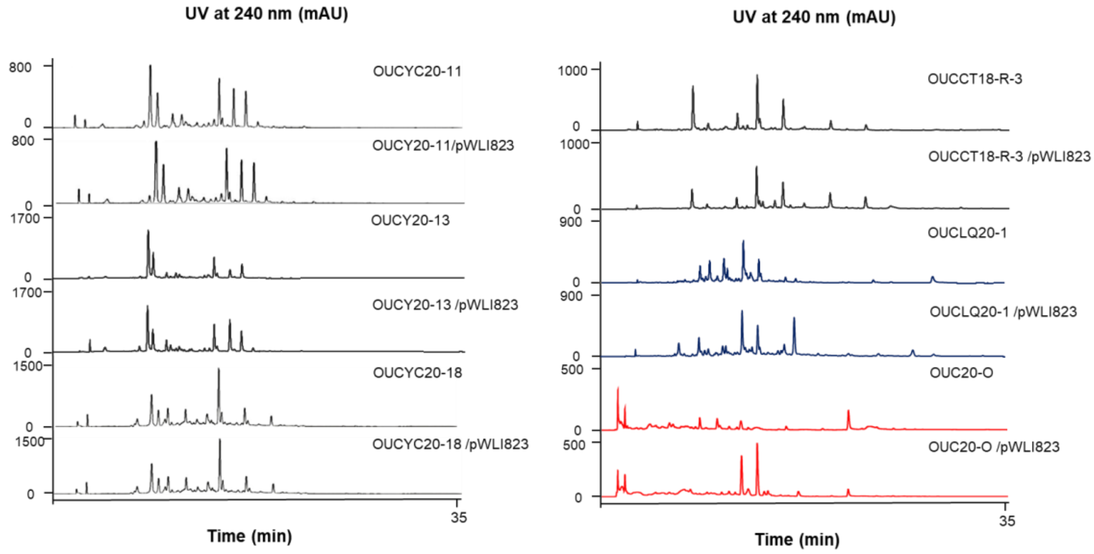

Figure S2. HPLC analysis of the culture extracts of six wild-type and recombinant strains of *Streptomyces* spp.

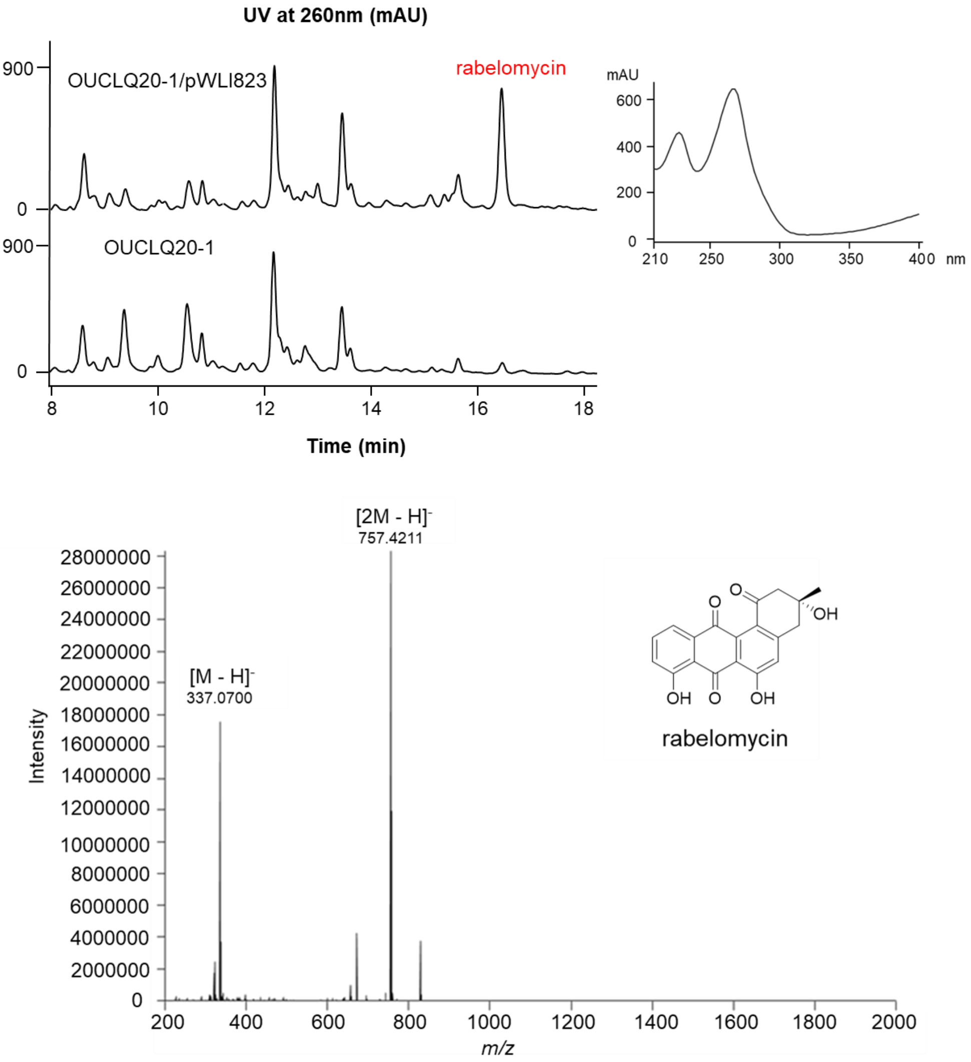

Figure S3. UV-HPLC-HRESIMS analysis of the activated rabelomycin in the recombinant strain of *Streptomyces* sp. OUCLQ20-1/pWLI823.

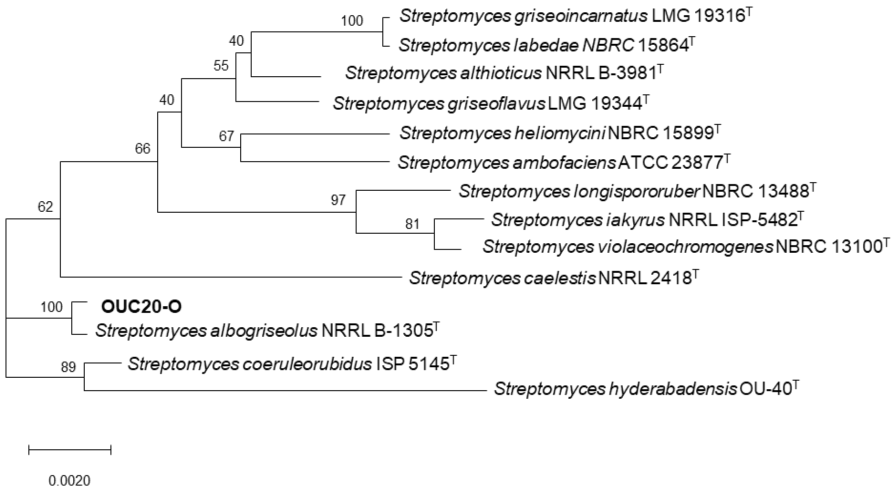

Figure S4. Neighbor joining phylogenetic tree based on 16S rRNA sequence of the OUC20-O strain.

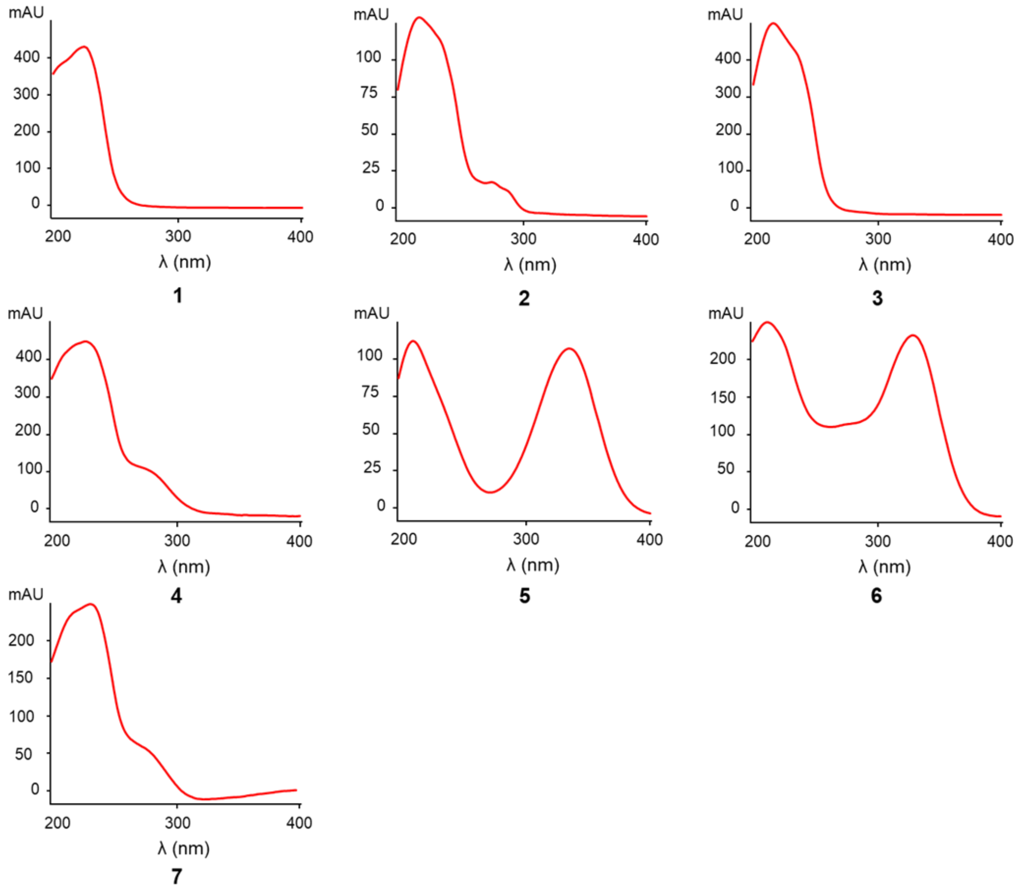

Figure S5. UV spectra of **1**−**7** in MeOH.

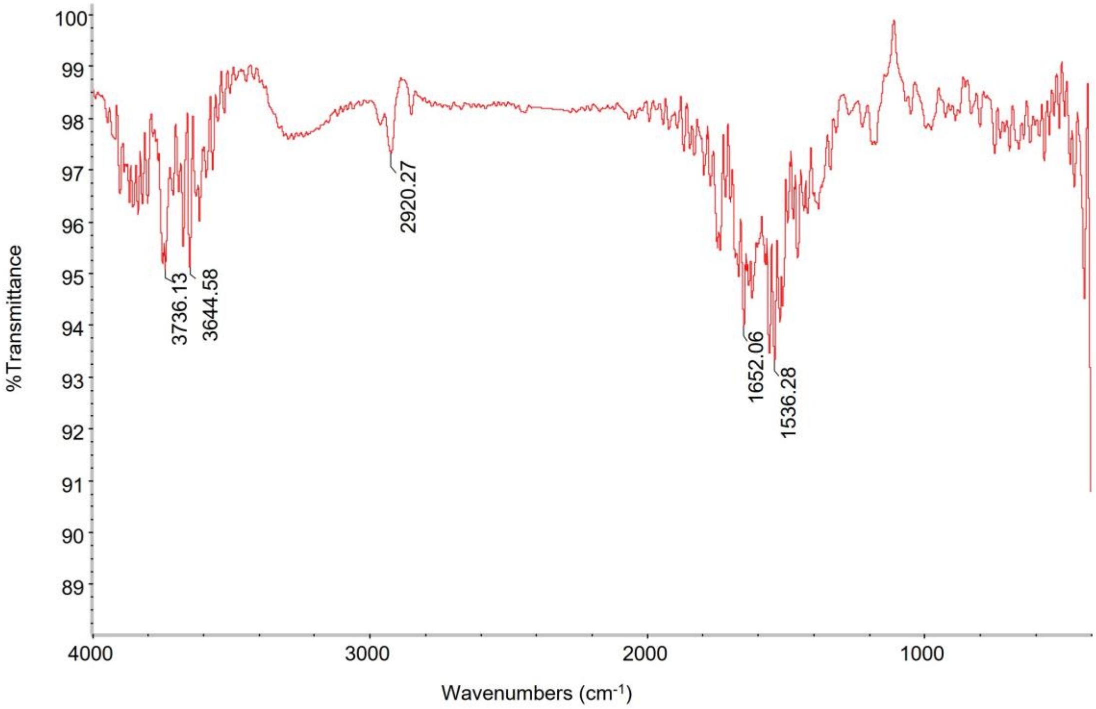

Figure S6. IR (KBr) spectrum of **1**.

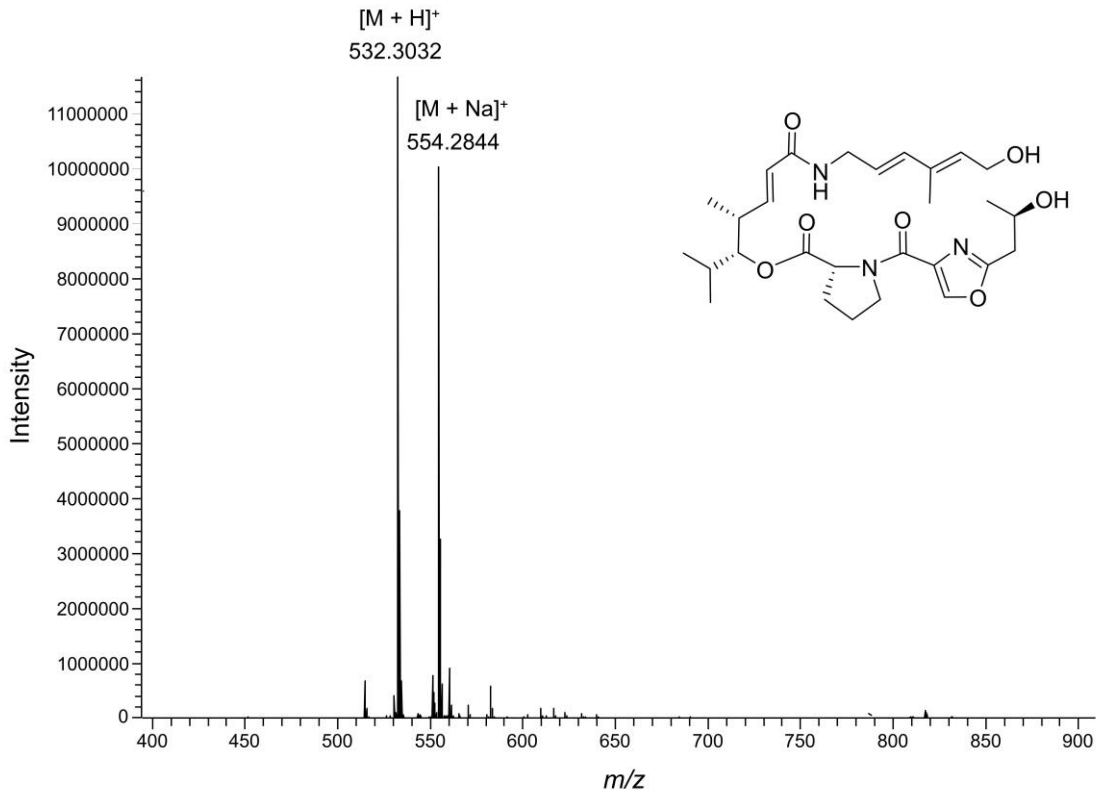

Figure S7. HRESIMS spectrum of **1**.

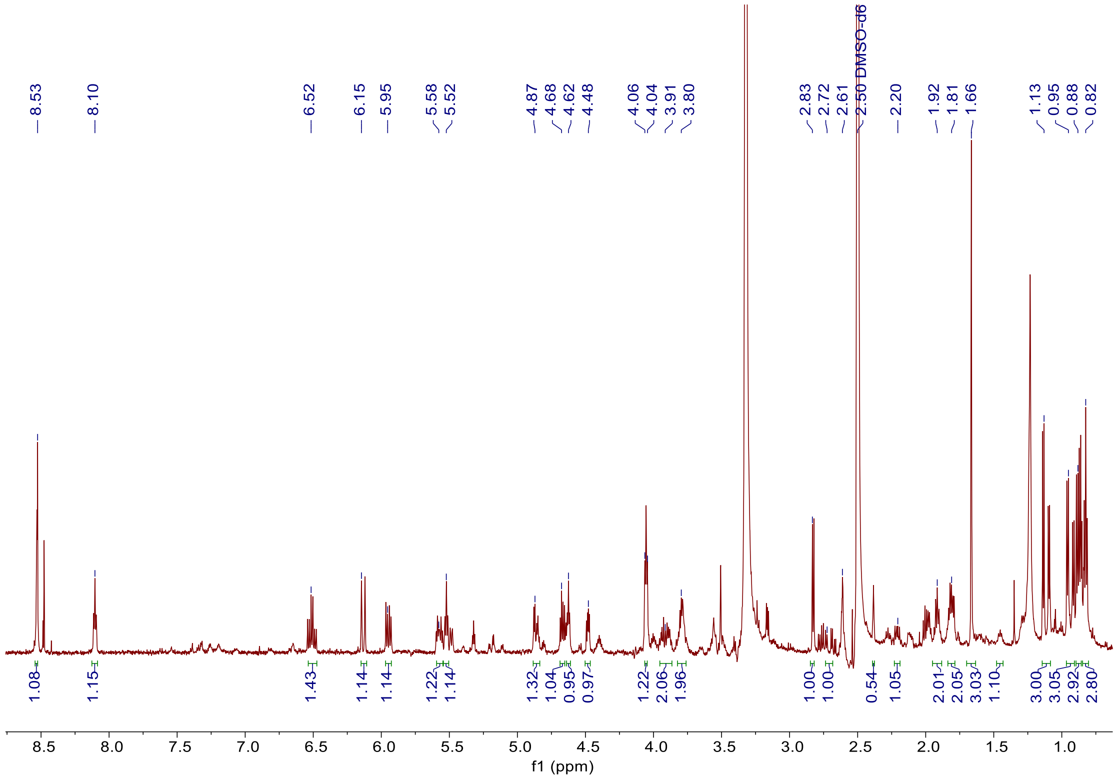

Figure S8. ^{1}H NMR spectrum of **1** in DMSO-d_6 (600 MHz).

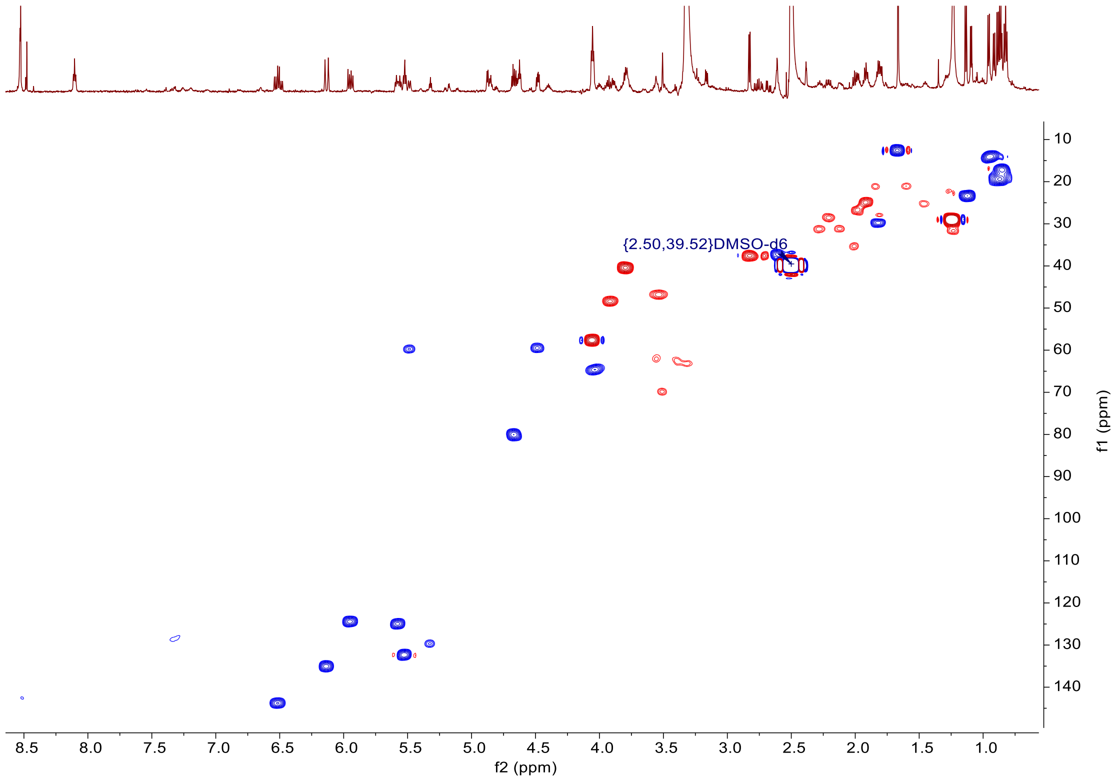

Figure S9. HSQC spectrum of **1** in DMSO-d_6 (600 MHz).

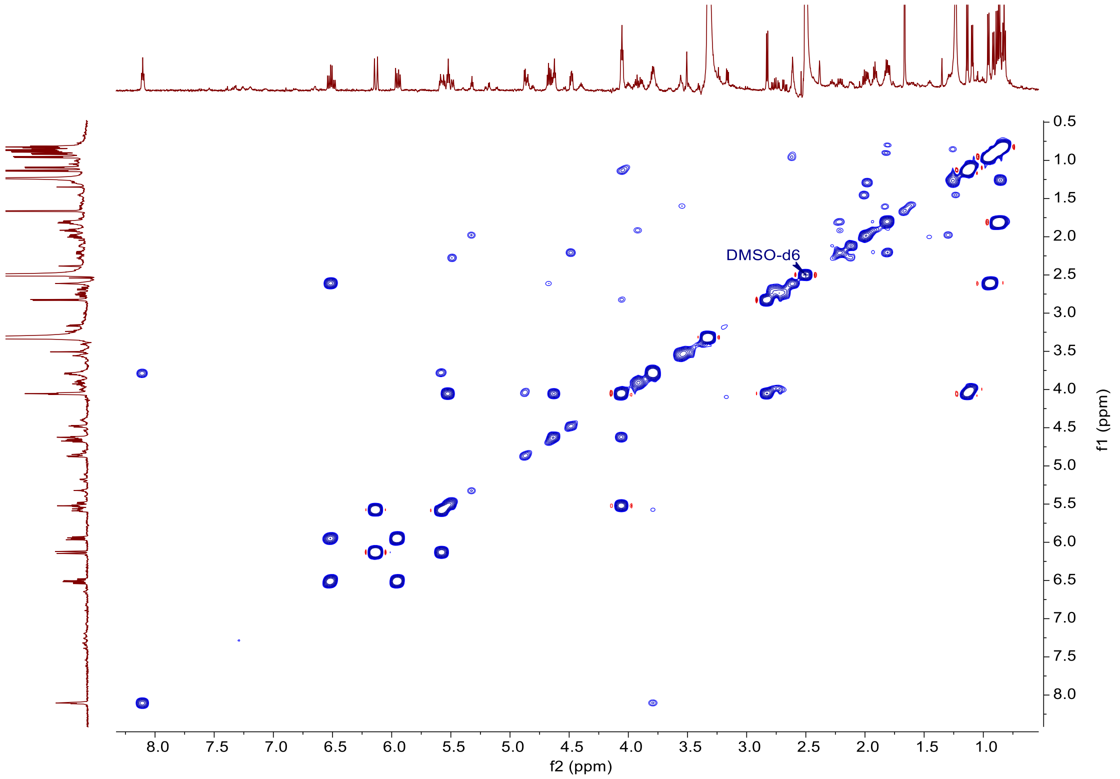

Figure S10 COSY spectrum of **1** in DMSO-d_6 (600 MHz).

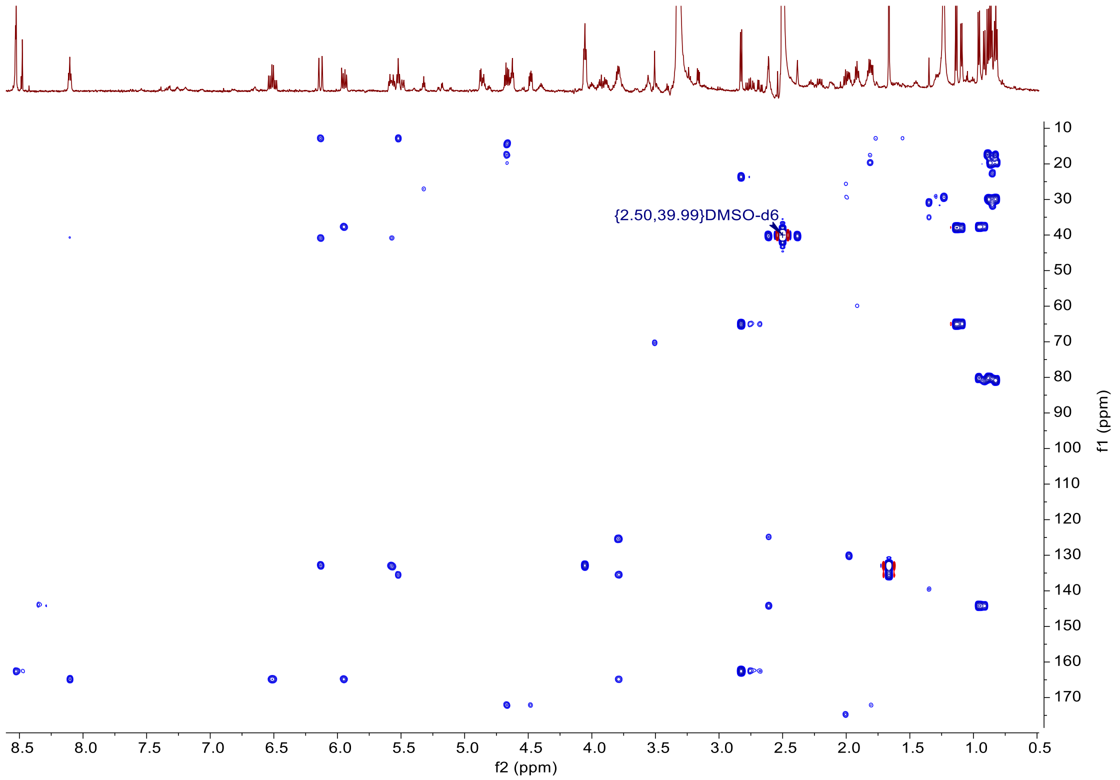

Figure S11. HMBC spectrum of **1** in DMSO-d_6 (600 MHz).

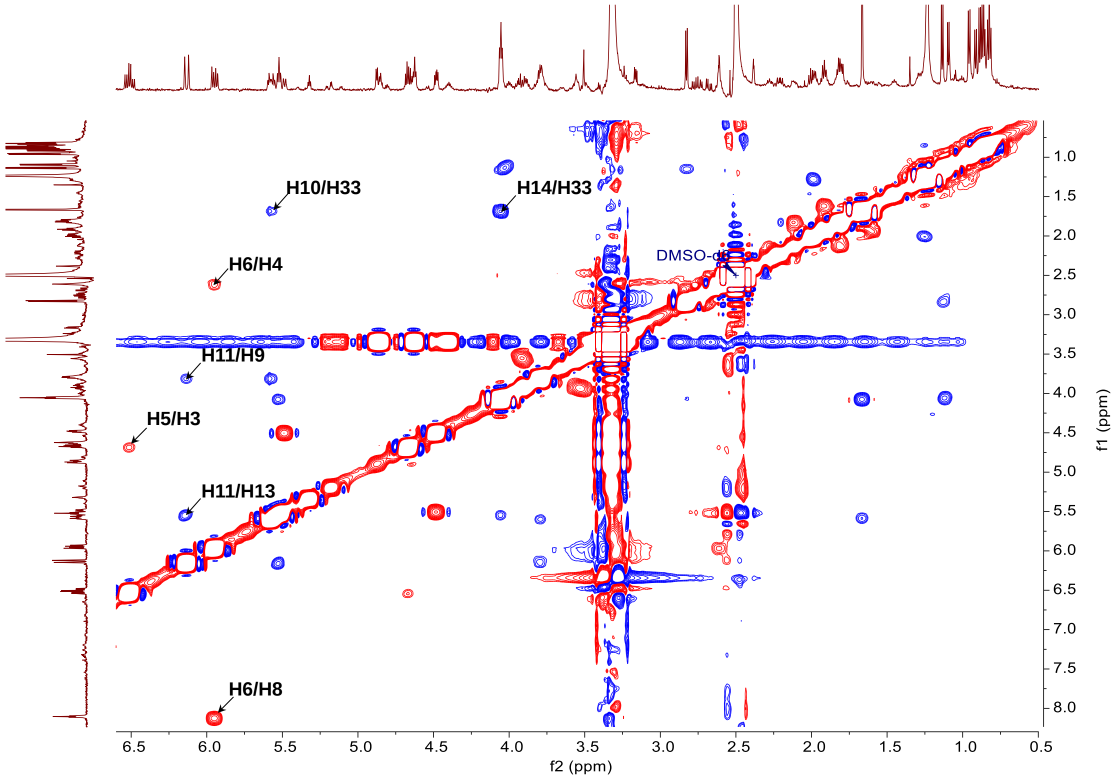

Figure S12. NOESY spectrum of **1** in DMSO-d_6 (600 MHz). Key NOE correlations were labeled.

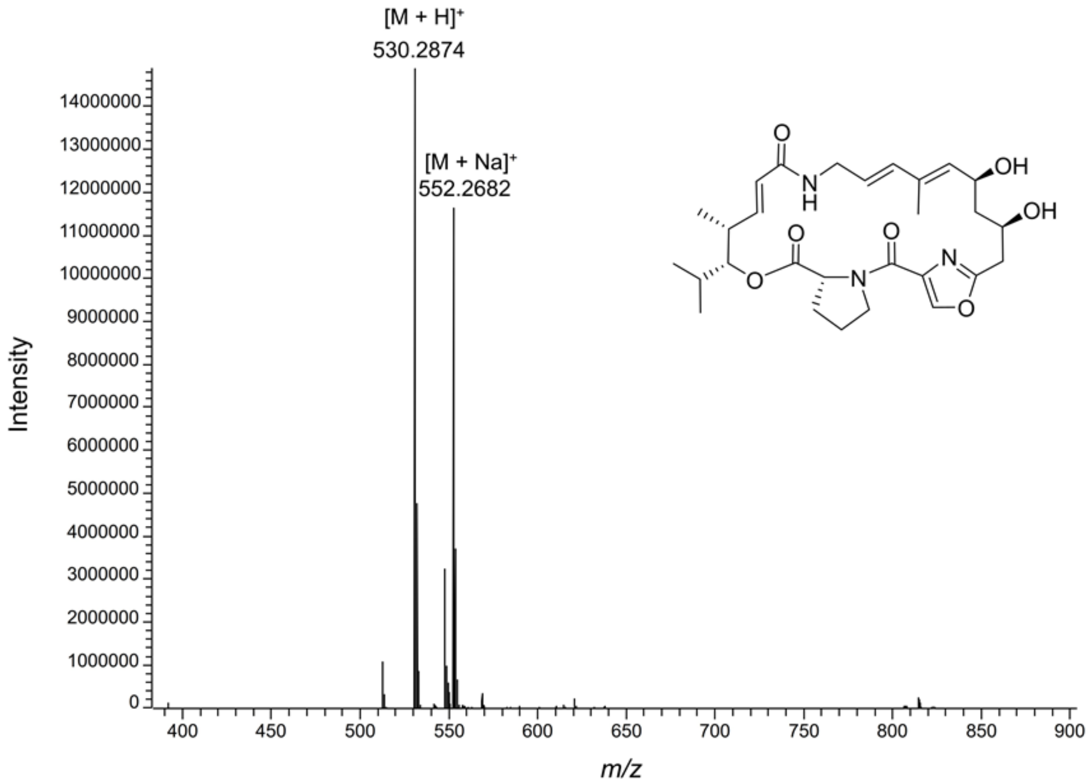

Figure S13. HRESIMS spectrum of **2**.

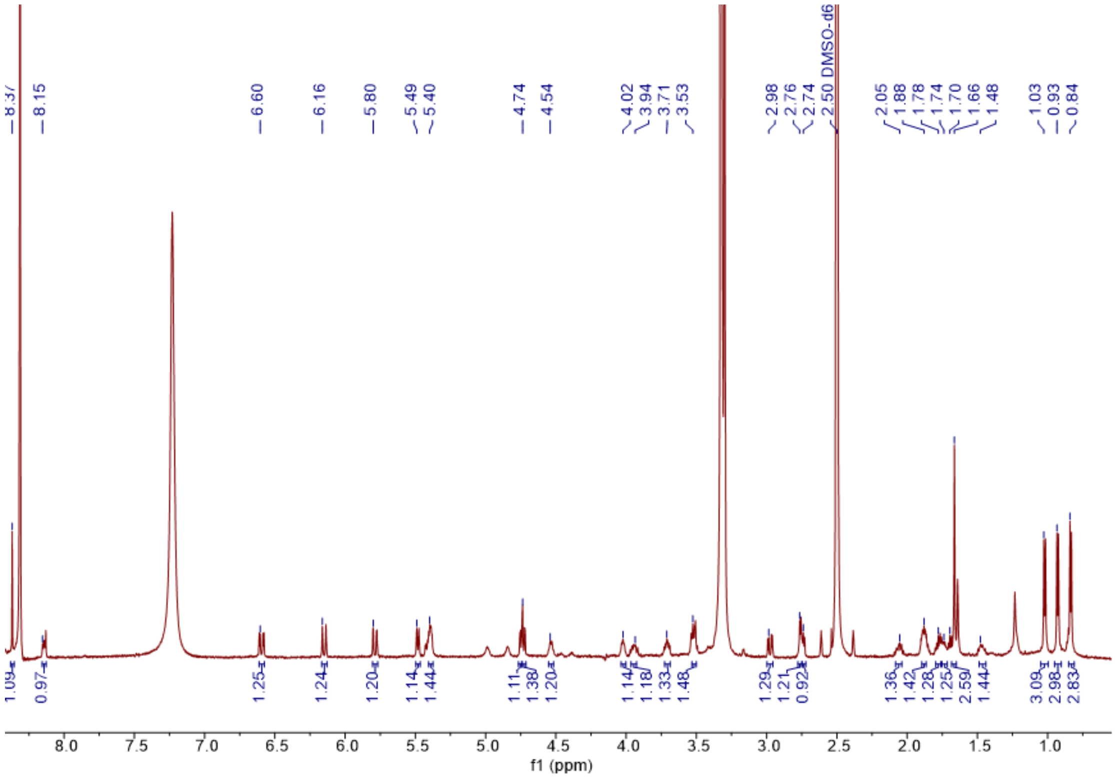

Figure S14. ^{1}H NMR spectrum of **2** in DMSO-d_6 (600 MHz).

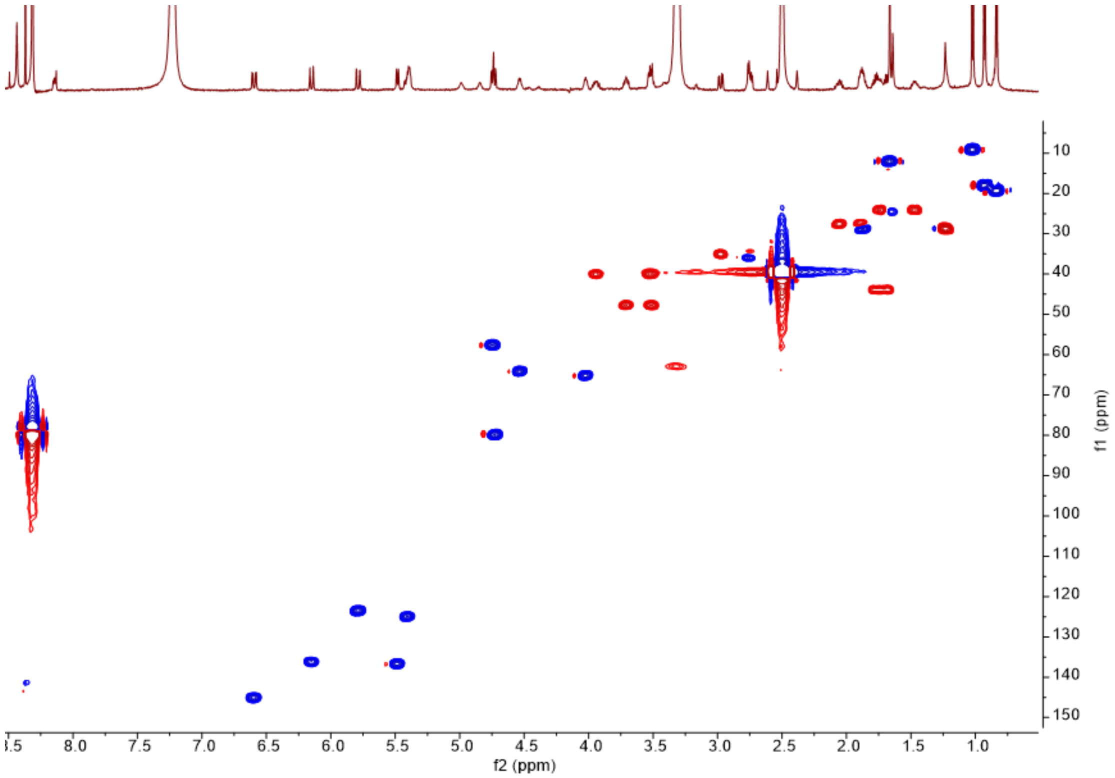

Figure S15. HSQC spectrum of **2** in DMSO-d_6 (600 MHz).

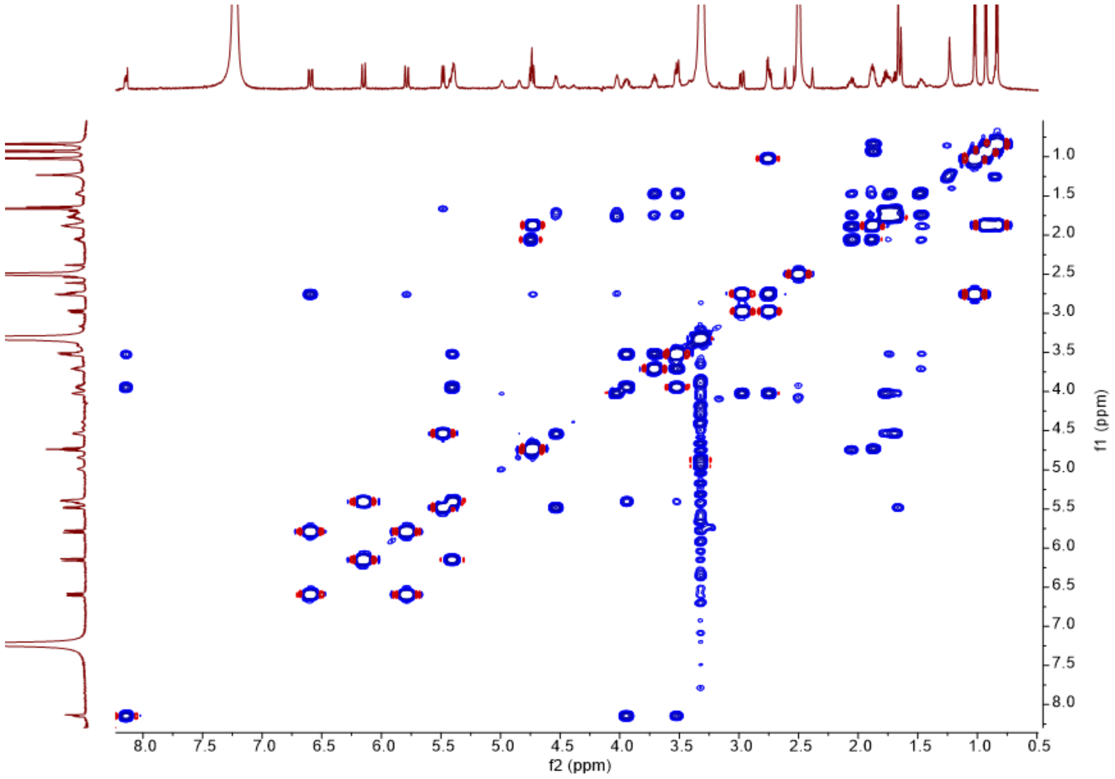

Figure S16 COSY spectrum of **2** in DMSO-d_6 (600 MHz).

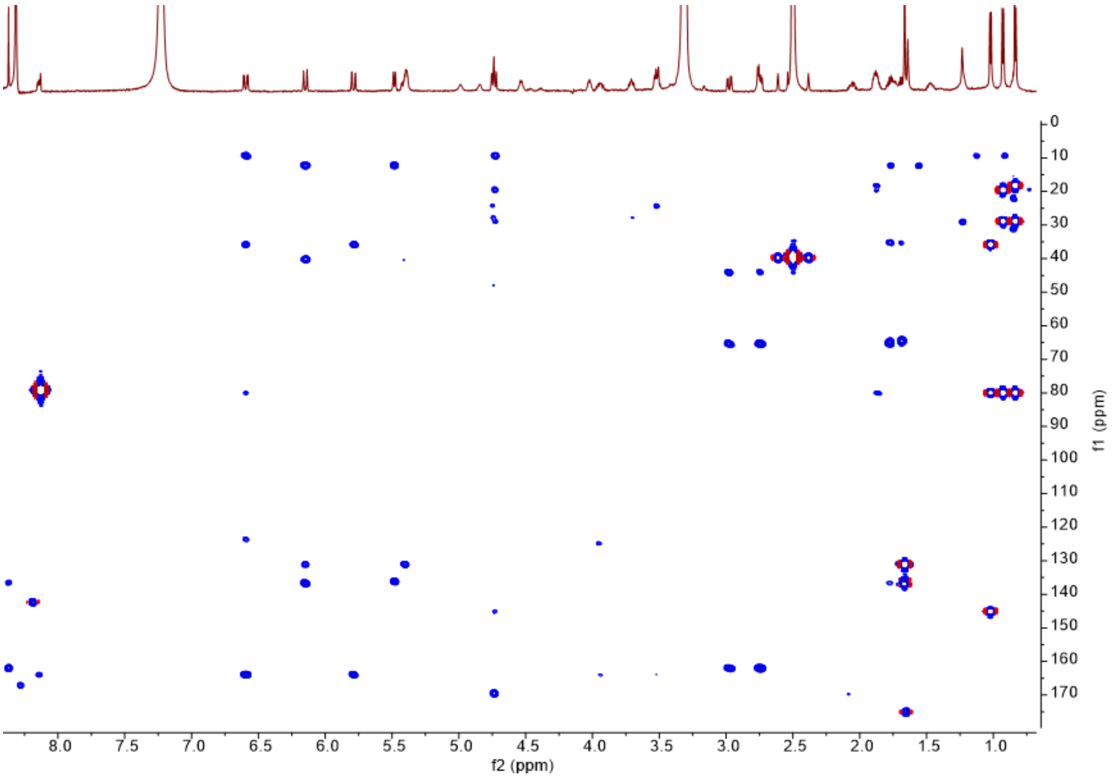

Figure S17. HMBC spectrum of **2** in DMSO-d_6 (600 MHz).

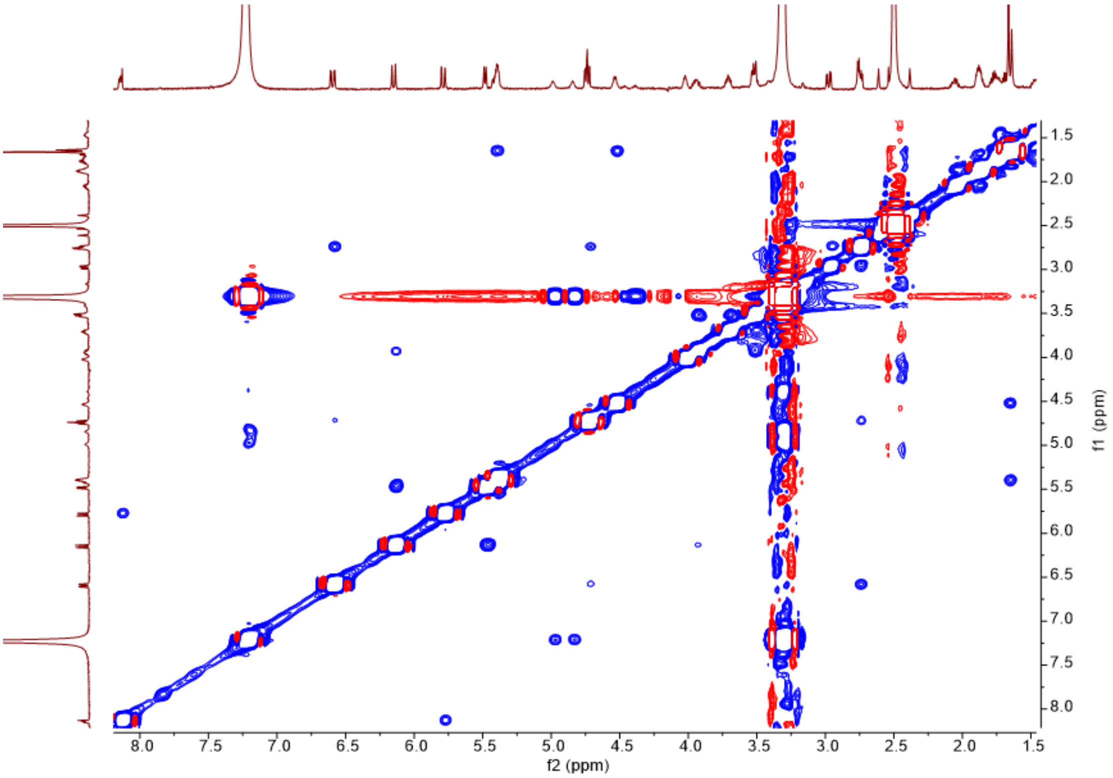

Figure S18. NOESY spectrum of **2** in DMSO-d_6 (600 MHz).

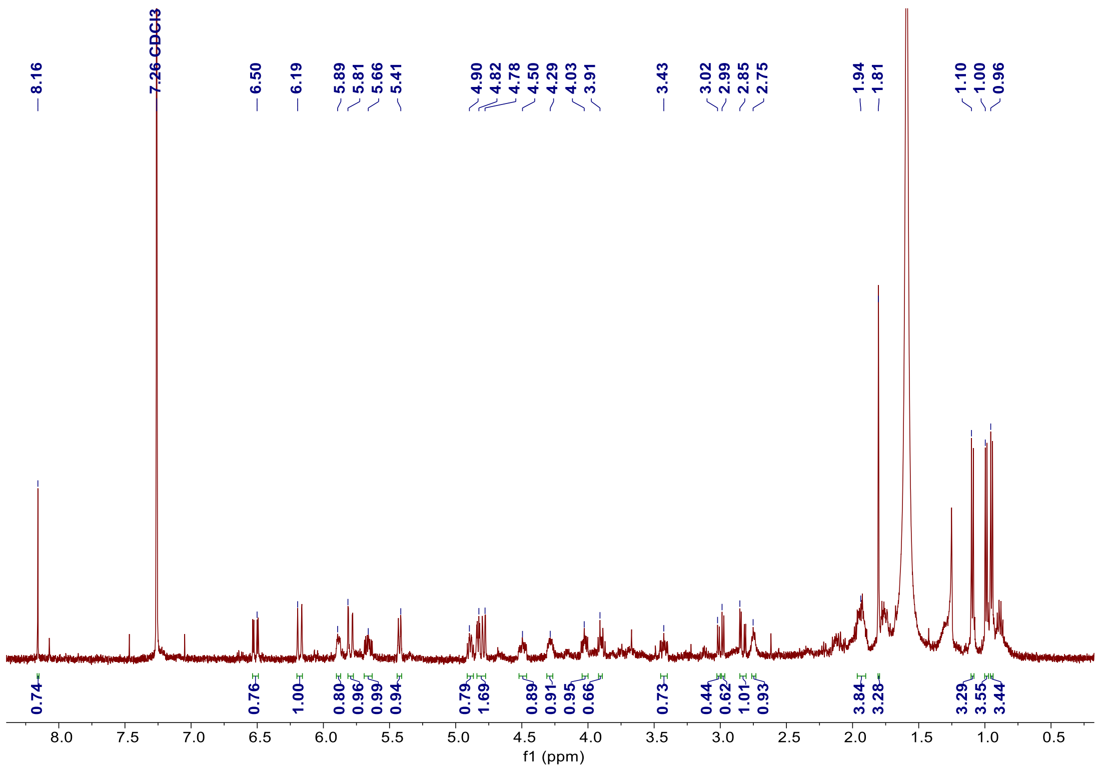

Figure S19. ^{1}H NMR spectrum of **2** in $CDCl_3$ (500 MHz).

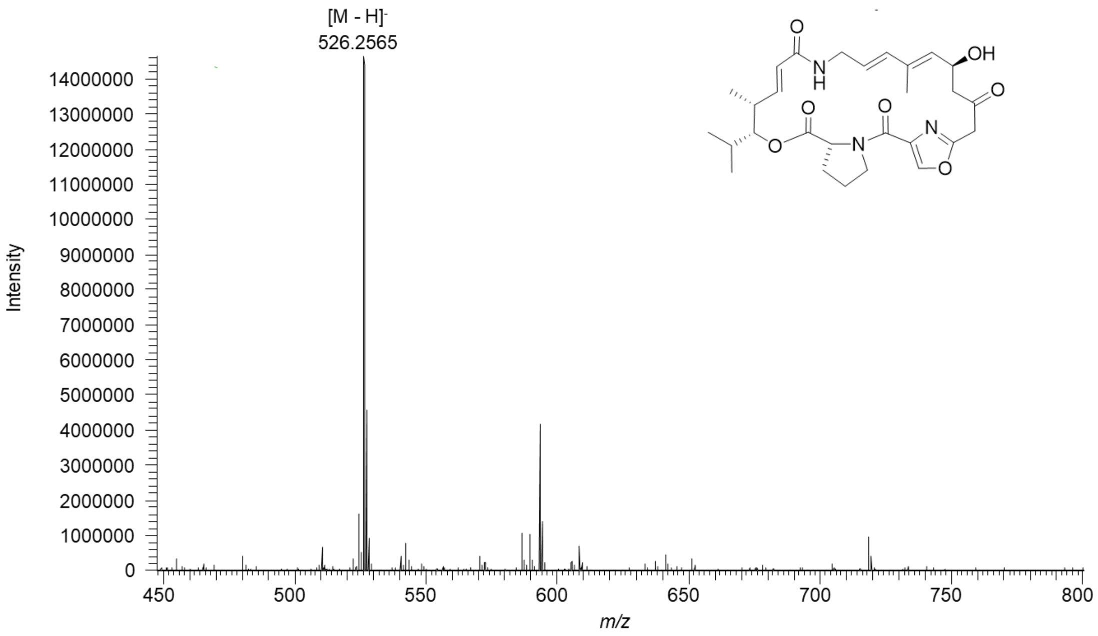

Figure S20. HRESIMS spectrum of **3**.

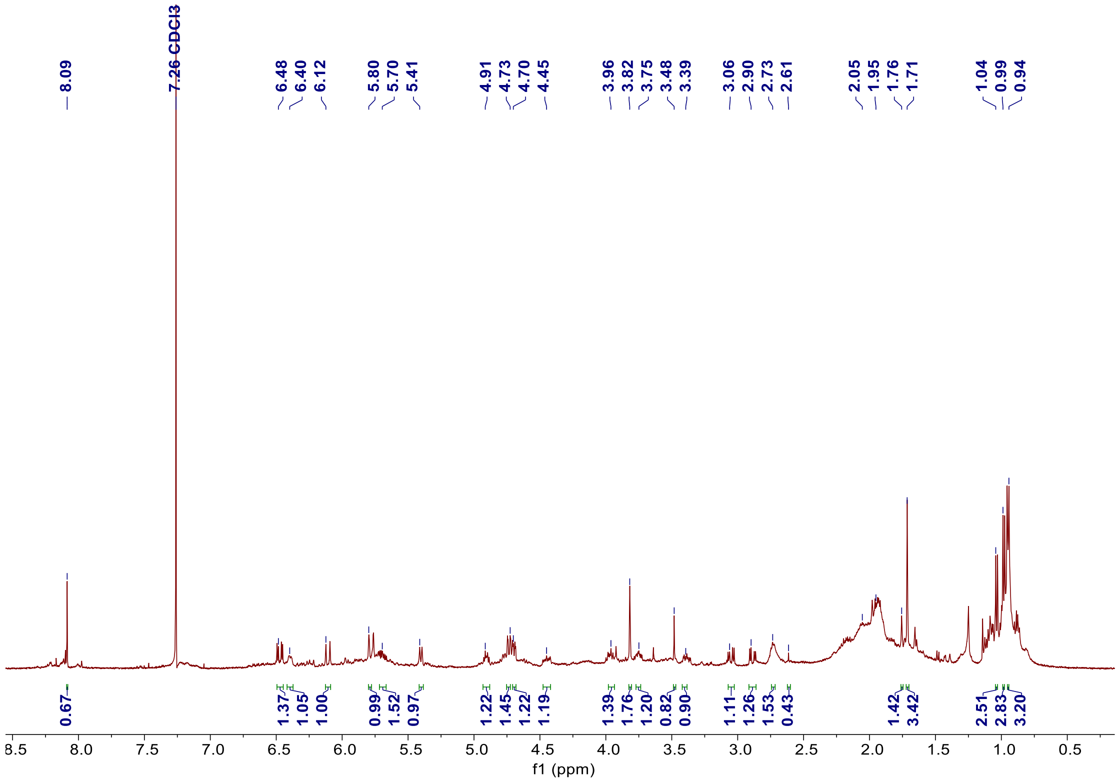

Figure S21. ^{1}H NMR spectrum of **3** in $CDCl_3$ (500 MHz).

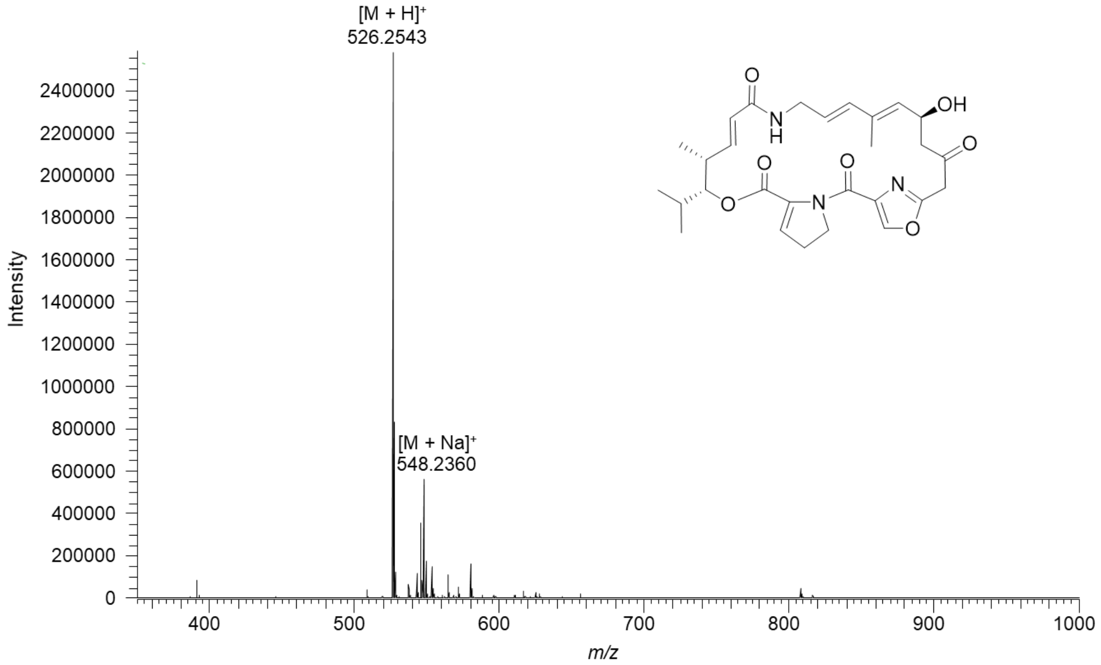

Figure S22. HRESIMS spectrum of **4**.

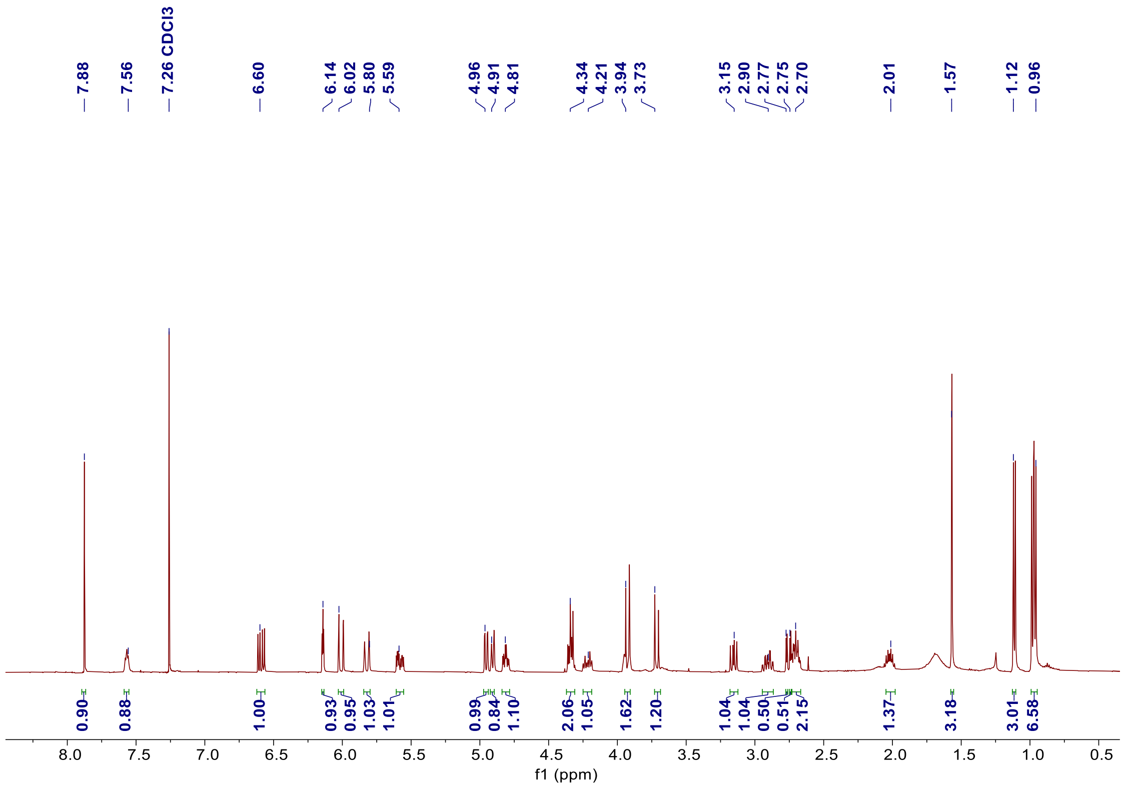

Figure S23. ^{1}H NMR spectrum of **4** in $CDCl_3$ (500 MHz).

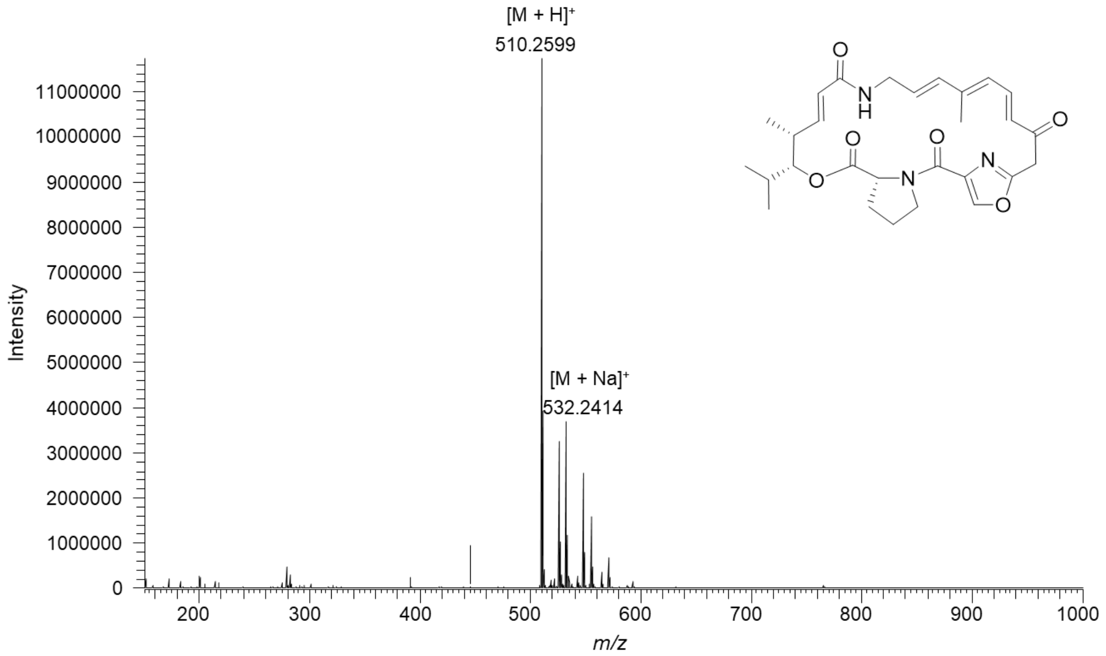

Figure S24. HRESIMS spectrum of **5**.

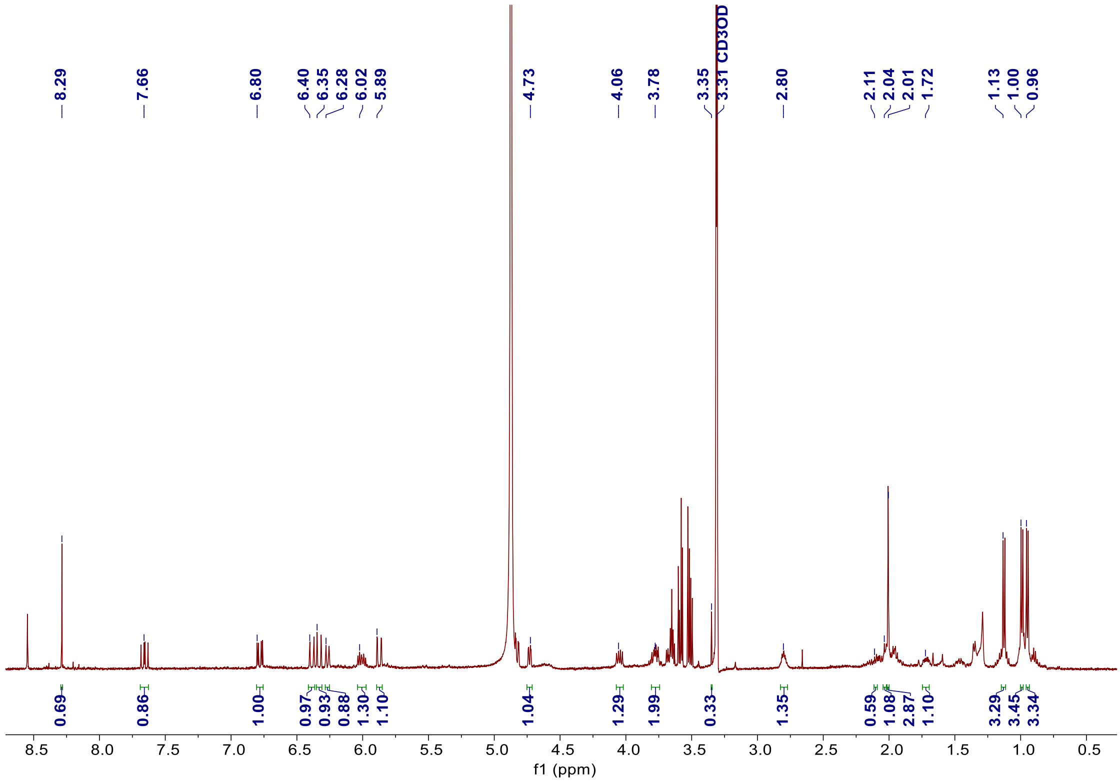

Figure S25. ^{1}H NMR spectrum of **5** in CD_3OD (600 MHz).

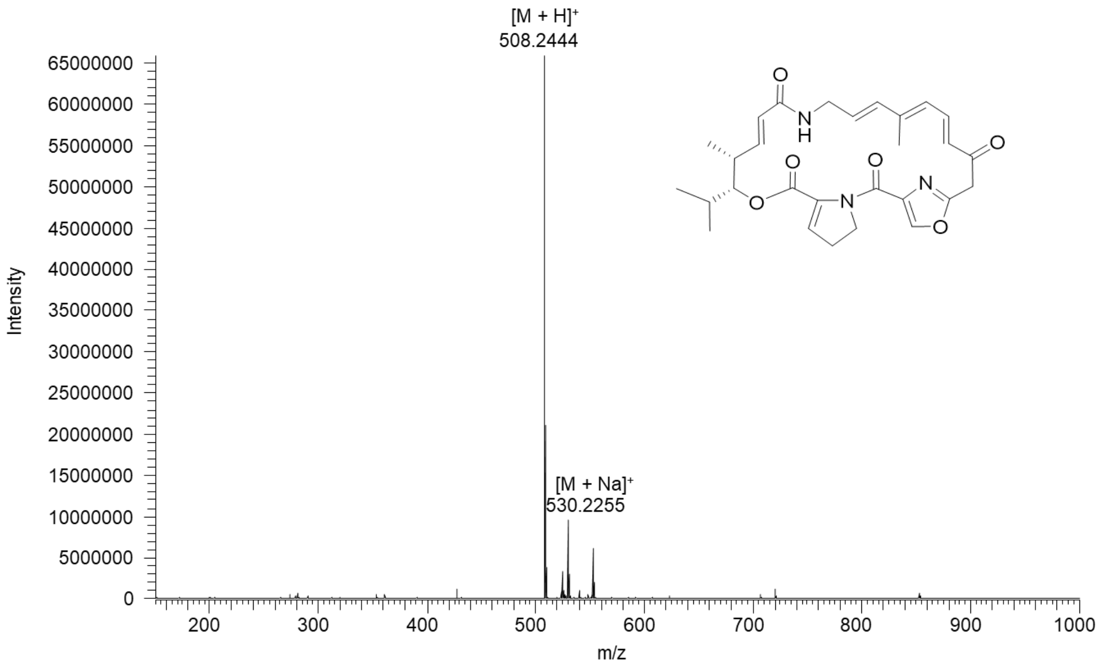

Figure S26. HRESIMS spectrum of **6**.

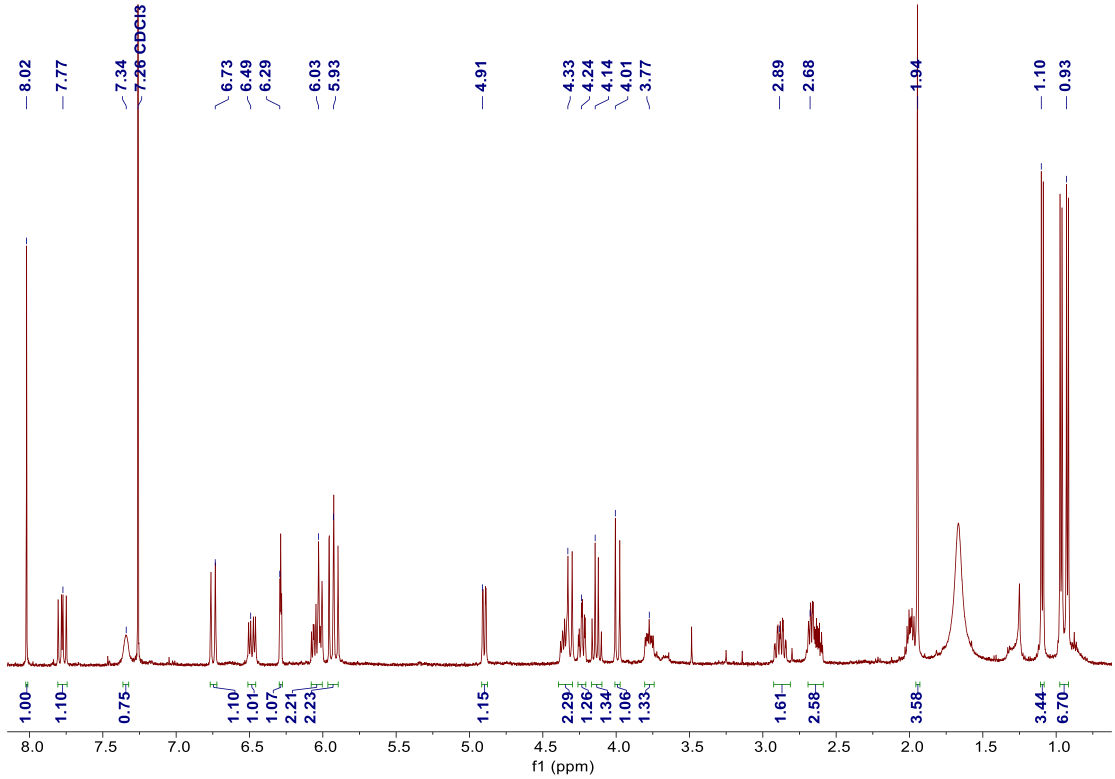

Figure S27. ^{1}H NMR spectrum of **6** in $CDCl_3$ (500 MHz).

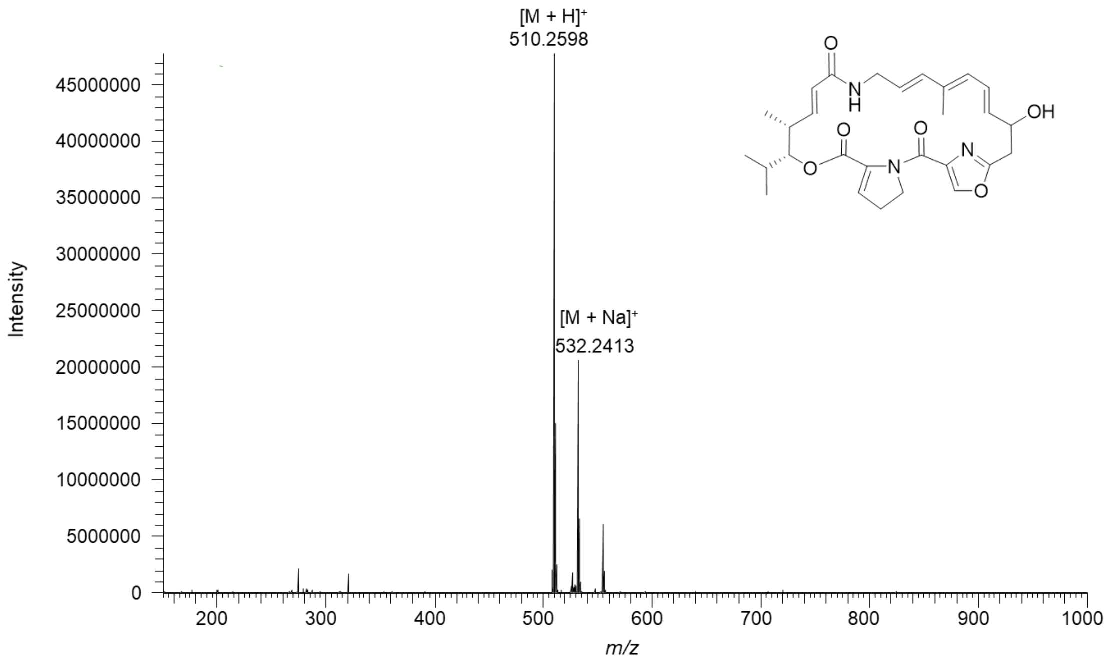

Figure S28. HRESIMS spectrum of **7**.

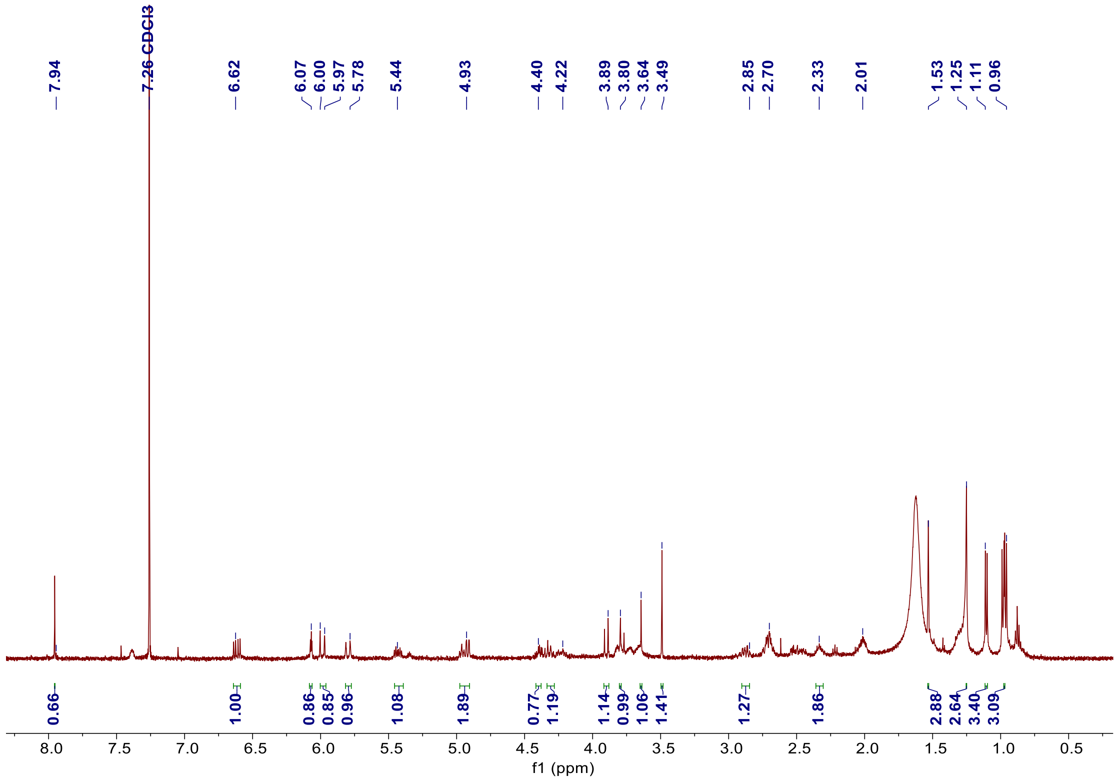

Figure S29. ^{1}H NMR spectrum of **7** in $CDCl_3$ (500 MHz).

Table S1. ^{1}H (600 MHz) and ^{13}C (150 MHz) NMR data of **2** DMSO-d_6.

	**2**	
No.	δ_C, type	δ_H (J in Hz)
1	169.5, C	-
3	79.8, CH	4.73, dd (9.7, 1.6)
4	36.0, CH	2.76, m
5	145.1, CH	6.60, dd (15.7, 4.0)
6	123.5, CH	5.79, d (15.8)
7	164.0, C	-
8	-	8.15, t (5.7)
9	40.0, CH_2	3.52, m
		3.94, m
10	124.9, CH	5.41, m
11	136.2, CH	6.15, d (15.4)
12	131.0, C	-
13	136.6, CH	5.49, d (8.7)
14	64.1, CH	4.54, m
15	43.9, CH_2	1.70, m
		1.78, m
16	65.2, CH	4.02, m
17	35.0, CH_2	2.74, dd (15.5, 5.7)
		2.98, dd (15.5, 4.7)
18	162.0, C	-
20	142.6, CH	8.37, s
21	136.5, C	-
22	161.8, C	-
24	47.7, CH_2	3.51, m
		3.71, m
25	24.1, CH_2	1.48, m
		1.74, m
26	27.6, CH_2	1.89, m
		2.06, m
27	57.6, CH	4.75, td (10.1, 3.0)
28	28.8, CH	1.87, m
29	19.4, CH_3	0.84, d (6.7)
30	18.0, CH_3	0.93, d (6.4)
31	9.1, CH_3	1.02, d (6.8)
33	12.0, CH_3	1.66, s
14-OH	-	4.84, s
16-OH	-	4.98, s

Table S2. DP4 calculated ^{1}H NMR data of **1**.

		Stereoisomer	
Position	Exp. **1**	16*R*-**1**	16*S*-**1**
3	4.67	4.52	4.56
4	2.61	2.82	2.84
5	6.55	6.42	6.70
6	5.95	5.93	5.78
9a	3.80	3.40	3.33
9b	3.80	4.11	4.17
10	5.57	5.87	5.84
11	6.15	6.35	6.31
13	5.53	6.00	5.84
14a	4.07	4.09	4.37
14b	4.07	4.27	3.92
15a	1.12	1.55	1.52
15b	1.12	1.21	1.18
15c	1.12	1.29	1.38
16	4.04	4.44	4.36
17a	2.82	2.97	2.99
17b	2.71	3.04	2.94
20	8.53	8.20	8.22
24a	3.91	3.87	3.93
24b	3.91	4.08	4.00
25a	1.92	2.02	2.02
25b	1.92	1.96	1.95
26a	2.21	2.34	2.27
26b	1.81	2.24	2.08
27	4.49	4.97	4.73
28	1.82	2.11	2.17
29a	0.87	1.38	1.54
29b	0.87	0.96	1.06
29c	0.87	0.71	0.72
30a	0.85	1.11	1.16
30b	0.85	1.06	1.11
30c	0.85	0.79	0.77
31a	0.94	0.87	0.98
31b	0.94	1.18	1.22
31c	0.94	1.03	1.12
33a	1.67	1.93	1.63
33b	1.67	1.60	1.85
33c	1.67	1.66	1.61
8-NH	8.11	6.18	5.86
14-OH	4.62	3.19	4.28
16-OH	4.86	4.25	4.25

Table S3. DP4 calculated ^{1}H NMR data of **2**.

Position	Exp. **2**	Stereoisomer	
		16*R*-**2**	16*S*-**2**
3	4.73	4.77	4.55
4	2.76	2.84	2.83
5	6.60	6.77	6.73
6	5.79	5.78	5.75
9a	3.94	4.36	4.25
9b	3.52	3.30	3.26
10	5.41	5.87	5.98
11	6.15	6.31	6.30
13	5.49	5.59	6.04
14	4.54	5.08	5.11
15a	1.78	2.06	2.72
15b	1.70	1.64	2.28
16	4.02	4.32	4.52
17a	2.98	3.06	2.88
17b	2.74	2.90	3.17
20	8.37	8.19	8.17
24a	3.71	4.19	4.10
24b	3.51	4.01	3.83
25a	1.74	1.88	1.95
25b	1.48	2.00	1.91
26a	2.06	2.09	2.03
26b	1.89	2.24	2.21
27	4.75	4.84	4.75
28	1.87	2.15	2.16
29a	0.84	1.05	1.06
29b	0.84	0.69	0.71
29c	0.84	1.44	1.53
30a	0.93	1.10	1.10
30b	0.93	1.13	1.15
30c	0.93	0.75	0.77
31a	1.02	1.28	1.22
31b	1.02	1.18	1.12
31c	1.02	1.03	0.98
33a	1.66	1.88	1.65
33b	1.66	2.03	2.00
33c	1.66	1.70	1.68
8-NH	8.15	5.27	5.66
14-OH	4.84	1.17	1.60
16-OH	4.98	2.66	3.90

Table S4. Cytotoxicity of compound **1** toward human normal cells.

Compound	Cell inhibition ±SD (%)		
	Concentration (μg/mL)	L-02[a]	293T[b]
**1**	20	1.93%±1.06%	7.99%±1.78%
	50	5.06%±1.84%	19.45%±0.46%
	100	8.52%±0.74%	23.72%±0.11%
Doxorubicin	10 μM	94.99%±0.33%	66.46%±0.94%

[a] Human hepatocytes; [b] Human embryonic kidney cells

Table S5. Primer pairs used in this study

gene	Primer pairs used for PCR confirmation of the strains (5'-3')
vioA	P_{gapDH}Xba IF: 5'-CTAGTCTACACGCACCCCCTGGTCAACGCG-3'
	vioA EcoR IR: 5'-CCGGAATTCTCACGCCGCCCAGACCCCAC-3'
16S rRNA	27F: 5'-AGAGTTTGATCCTGGCTCAG-3'
	1492R: 5'-GGTTACCTTGTTACGACTT-3'

Table S6. Strains used in this study

Name	Top-hit taxon	Top-hit strain	Similarity (%)
OUCYC20-11	*Streptomyces griseoincarnatus*	LMG 19316	99.58
OUCYC20-13	*Streptomyces tunisiensis*	CN-207	99.35
OUCYC20-18	*Streptomyces rochei*	NRRL B-2410	99.15
OUCT18-R-3	*Streptomyces olivaceus*	NRRL B-3009	99.50
OUCLQ20-1	*Streptomyces ginkgonis*	KM-1-2	99.75
OUC20-O	*Streptomyces albogriseolus*	NRRL B-1305	99.79
OUCYC20-12	*Streptomyces thermolineatus*	DSM 41451	99.75
OUCYC20-14	*Streptomyces pratensis*	ch24	100
OUCYC20-15	*Streptomyces griseoflavus*	LMG 19344	99.41
OUCYC20-16	*Streptomyces violascens*	ISP 5183	99.15
OUCYC20-17	*Nocardia prasina*	DSM 43845	99.50
OUCYC20-19	*Streptomyces nigra*	452	99.51
OUCYC20-20	*Streptomyces violascens*	ISP 5183	99.15
OUCYC20-21	*Streptomyces carpaticus*	NBRC 15390	98.94
OUCYC20-22	*Streptomyces albogriseolus*	NRRL B-1305	99.30
OUCYC20-23	*Streptomyces xiamenensis*	MCCC 1A01550	99.09
